# Atypical neural responses to dynamically changing facial expressions correlate with behavioral risk in toddlers born preterm: Evidence from an ERP study

**DOI:** 10.1016/j.ynirp.2026.100333

**Published:** 2026-03-22

**Authors:** Johanna Bick, Xinge Li, Haley Laughlin, Andrea Jimenez Ortiz, Anna Galvan, Kelly Vaughn, Cara Price, Susan Landry, Dana DeMaster

**Affiliations:** a University of Houston, USA; b University of Texas Health Science Center, USA

**Keywords:** Prematurity, Neurodevelopment, Facial emotion processing, ERPs, Socioemotional risk

## Abstract

Premature birth increases lifelong risk for emotional dysregulation and mental health problems. Identifying alterations in neural pathways that subserve social cognitive development may help identify variability of prospective risk on behavioral levels. In a sample of 45 toddlers (15–30 months corrected age) born prematurely (23–36 gestational weeks; extremely preterm: n = 21, very preterm: n = 13, late preterm: n = 11), we leveraged EEG as a developmentally sensitive neuroimaging tool, and examined associations between premature birth status and event related potentials (ERPs; N290, P400 and NC) elicited while toddlers passively viewed dynamically changing facial emotion expressions. Using linear-mixed effects models, earlier gestational age was associated with larger P400 and frontal NC amplitudes and with differences in the latencies and hemispheric organization of ERP responses to facial emotion (FDR-corrected p < 0.05). Specifically, toddlers born at earlier gestational ages showed reduced right to left hemispheric differentiation in N290 latency, reflecting diminished rightward specialization, along with emotion dependent differences in P400 and NC latencies, consistent with a less mature profile of social brain development. Source localization techniques pointed to frontal and temporal regions as neural generators of ERP components, which supports prior work showing heightened vulnerability of these circuitries to effect of prematurity. Brain-behavioral correlations support preliminary associations between ERPs and early childhood psychopathology risk, highlighting the potential to leverage neural markers to inform prevention and intervention.

## Highlights

• Early gestational status was associated with atypical P400 and frontal NC amplitudes and N290 and central NC and latencies.
• The most prematurely born children displayed reduced specialization and discrimination of emotional faces.
• Source localization techniques pointed to frontal and temporal cortical regions as neural generators of ERP variability.

## 1 Premature birth is associated with neural substrates of facial emotion processing and behavioral risk in toddlerhood

Premature birth is long shown to increase risk for cognitive, academic, and mental health problems across the lifespan (Wilson-Costello et al., 2005). Even in the absence of severe medical complications or brain injury, over half of all prematurely born children experience socio-emotional difficulties (Dean et al., 2021; Janvier et al., 2012; Ritchie et al., 2015; Rogers and Hintz, 2016) and remain at increased risk for a clinically elevated mental health problems at some point across the lifespan (Fenoglio et al., 2017). Due to increased survival rates of babies born at earlier gestational stages, there has been a dramatic increase in prevalence rates for neuropsychiatric disorder that emerge in childhood and beyond (Chawanpaiboon et al., 2019). Understanding signatures of socio-cognitive risk and underlying neurodevelopmental mechanisms, may have translational value for developing maximally effective prevention and intervention that serve this vulnerable population.

**Emotional Face Processing.** Human social development is a protracted process that begins in early infancy (perhaps even rudimentarily in utero) and unfolds in the context of early social relationships. Social signals conveyed by caregivers serve as formative input that fine tunes the social brain. Newborns show a preference for looking at faces versus objects (Pascalis et al., 1995), and develop preferential attention to caregiver faces as their visual system matures. This preferential attention facilitates formation of attachment relationships and scaffolds social development in the first months of life. Babies born prematurely are more likely to show delays in the development of social brain circuitries, that support foundational abilities to recognize social cues from facial emotions. According to meta-analytic work of 8 studies, premature birth associates with poorer facial emotion recognition skills from preschool to adult years. Birth earlier than 28 weeks (the clinical cut off for very/extreme stages of premature birth) is associated with higher behavioral risk. Prematurity related disparities in facial emotion recognition appear to be most prominent in adolescence, which is a well-established sensitive period for the onset of mental health disorders (Valderrama Yapor and Nosarti, 2025).

There is a paucity of work investigating gestational risk prior to preschool stages of development, highlighting a key gap in understanding of early origins of risk. This is especially surprising given the breadth of literature on normative development of face processing involving typically developing infants. However, the understanding of typical development serves as an important foundation from which to test atypical development trajectories secondary to prematurity birth. According to seminal work, prior to six months of age, infants show a visual preference toward happy versus angry facial expressions (Barrera and Maurer, 1981), often described as an early “positivity bias”. By 7 months of age, the pattern appears to reverse: infants show a visual preference to fearful versus happy facial expressions (Kestenbaum and Nelson, 1990), often referred to as a “negativity bias”, which may reflect increased attention allocation and less visual expertise with adults' fearful expressions at this stage of life. Abilities to discriminate positive facial expressions (happy, surprise) from negative facial expressions (fear, anger, sadness) is shown to emerge by 10 months of age (Ludemann, 1991). By 12 months, infants show greater discrimination between negative emotions (fear and anger) and begin to use adults’ expressions to guide their actions and behavior (Baldwin and Moses, 1996; Grossmann et al., 2007; Vaish et al., 2008).

**Neural Substrates of Emotional Face Processing.** Neural substrates of face processing have been widely investigated in the first years of life using Electroencephalography (EEG). EEG is more tolerant of motion than other neuroimaging methods, making it well suited for infant populations. EEG also provides excellent temporal resolution capturing neural responses on the millisecond level, which serves as a relative advantage over MRI or other modalities. Decades of research has revealed a reliable set of event related potentials (ERPs; time-locked EEG responses) that emerge in infancy and are sensitive to face familiarity and emotion expression. The N290 and the P400 components support perceptual processing of facial structure (De Haan and Nelson, 1999; Guy et al., 2016; Haan et al., 2002; Halit et al., 2003, 2004; Key et al., 2009; Xie and Richards, 2016) and are considered a developmental precursor to the face sensitive N170 component that appears in early childhood (Halit et al., 2003). The N290 component peaks around 290 ms and has maximal amplitude over the posterior parietal-occipital cortex, while the P400 ERP component is a positive deflection that peaks between 350 and 500 ms and shows maximal amplitudes at occipital regions. Prior work has reliably shown the N290 to be localized to the fusiform-gyrus and superior-temporal sulcus that support face processing and other domains of social cognition (Guy et al., 2016; Halit et al., 2004; Leppänen et al., 2007; Sadeh et al., 2010). The P400 has been localized to the midline frontal and parietal, anterior temporal, and posterior temporal and occipital regions (Guy et al., 2016).

Longitudinal studies show that children begin to show neural discrimination in the N290 and P400 components to faces versus non faces starting around 3 months of age (De Haan et al., 2013). Some work shows that these components discriminate positive, negative, and neutral facial emotion expressions around 5 to 7 months of age (De Haan and Nelson, 1999; Haan et al., 2002; Halit et al., 2003; Leppänen et al., 2007). Some studies report larger N290 and P400 amplitudes to fearful versus non-fearful (happy, angry, and neutral) facial expressions (Leppänen et al., 2007; Aran et al., 2023; Hoehl and Striano, 2008; Xie et al., 2019; Yrttiaho et al., 2014) although other studies do not support this bias (Jessen and Grossmann, 2017; Rajhans et al., 2016; Van Den Boomen et al., 2019; Vanderwert et al., 2015). Parental risk factors and attachment insecurity may attenuate N290 bias toward fearful faces (Peltola et al., 2020); thus lack of consideration of contextual factors may contribute to heterogeneity in findings across the literature. For instance, some studies report larger N290s to happy versus non-happy faces in infants around 7 months of age (Van Den Boomen et al., 2019; Quadrelli et al., 2019), while others suggest larger N290 amplitudes to angry versus fearful faces, suggesting that cues of threat may be particularly salient in infants (Kobiella et al., 2008), although this is not supported in all work (Aran et al., 2023). P400 sensitivity to threatening emotional facial expressions (angry and fearful faces) has also been reported in several studies (Hoehl and Striano, 2008; Xie et al., 2019; Kobiella et al., 2008) infants reported as having higher negative affect have shown larger P400s to negative facial expressions (Quadrelli et al., 2019).

The NC is a negative deflection peaking between 300 and 600 ms with maximal amplitude over frontal and central regions. The NC has been considered to reflect an obligatory attentional response to salient, meaningful, or novel stimuli (De Haan et al., 2013) and has been localized to frontal and anterior-cingulate cortex regions that support top-down control (Guy et al., 2016; Reynolds and Richards, 2005). Seminal work indicates that by 7 months of age, infants show relatively larger NC amplitudes to fearful versus happy faces (Nelson and De, 1996; Peltola et al., 2009), reflecting increased attentional resources a facial emotion expression that is perhaps less familiar at this stage of life. Other studies of 7-month-old infants shows a larger NC to angry faces versus negatively-valenced or neutral faces (Hoehl and Striano, 2008; Xie et al., 2019; Kobiella et al., 2008), suggesting abilities to differentially allocate attention to negatively-valenced facial expressions at this early stage in development. Contrasting other work, one study reported larger NCs to happy versus angry (Grossmann et al., 2007) and fearful facial expressions have been reported (Van Den Boomen et al., 2019), raising questions about the need to further explore contextual factors or methodological characteristics that influence variability NC differences observed across studies. Few studies have examined ERP differences following 12 months of age. Two existing studies have compared typically developing toddlers with those exposed to extreme early life adversity or stress in family settings. Infants reared in healthy family environments showed relatively larger NC amplitudes to angry faces versus happy faces or neutral faces; those exposed to extreme stress showed the opposite trend (Curtis and Cicchetti, 2013). In another study, infants and toddlers exposed to extreme psychosocial neglect showed smaller NC amplitudes to faces of varying positive and negative emotions, relative to children reared in family settings. Data also suggest that NC amplitudes decreased as a function of age; younger infants showed larger NC amplitudes relative to older infants and toddlers (Parker and Nelson, 2005).

Most ERP studies examine neural responses to static images of facial expressions, yet there is growing interest in the use of more ecologically valid paradigms that may better capture neural and behavioral processes such as experienced on a day-to-day bases(Barrett et al., 2019). To our knowledge, only two studies have reported on ERPs to display dynamically changing facial expressions in infancy (and non in toddlers) (Quadrelli et al., 2019; Missana et al., 2014). Both studies involved typically developing infants around 7 months of age. In one study, infants passively viewed facial expressions transitioning from neutral to painful expressions and neutral to angry expressions (Missana et al., 2014). Results from this study showed that NC amplitudes were larger to faces transitioning from neutral to painful expressions, than faces that transitioning from neutral to angry expressions (Missana et al., 2014). In the second study, infants passively viewed faces transitioning from neutral to angry, neutral to happy, or remaining neutral during the stimulus presentation (Quadrelli et al., 2019). In this study, P400s were larger to the happy (but not angry) condition versus the neutral condition./Further, infants showed larger NCs to faces transitioning from neutral to happy and neutral to angry expressions relative to the neutral condition; these differences were specific to the right hemisphere, which is consistent with typical development of right hemispheric specialization for face processing (Quadrelli et al., 2019).

NC amplitudes have been associated with behavioral and emotional risk in samples of typically and atypically developing samples of young children. Some work supports connections between parental responsiveness and contextual factors (Taylor‐Colls and Pasco Fearon, 2015). In one study, 7-month-old infants with temperaments traits of better emotion regulation showed larger right hemisphere NC amplitudes to fearful versus happy faces; infants with temperamental traits involving more negative emotionality showed the opposite direction of effects (Martinos et al., 2012). In another study, infants with more “positive” temperamental factors showed larger NCs to fearful versus happy faces; infants with more “fearful” temperaments show similar patterns, but stronger right hemisphere lateralization (De Haan et al., 2004). Slower NCs latencies (regardless of facial emotion expression) have also correlated with greater risk for emotion dysregulation (Martinos et al., 2012). Thus, the NC ERP component may be an especially sensitive marker of socio-emotional risk.

**Prematurity and Neural Substrates of Facial Emotion Processing.** There is emerging evidence that premature birth interferes with abilities to recognize and infer social cues from facial expressions, yet research on neural substrates of risk is limited. We know of two studies that have investigated neural function in association with face processing in children who vary in premature birth status. One study used Magnetencephalography (MEG), which measures neural oscillation similar to EEG. MEG was recorded in school age children (between 6 and 8 years) during an implicit emotional face processing task. Children born prematurely showed poorer performance on a task of face recognition (based on greater attribution errors and likelihood of mis-construal), but they showed significantly different network connectivity dynamics in social brain region relative to full term controls (Mossad et al., 2020). We know of only one EEG study that has investigated connections between premature birth and neural function associated with face processing. EEG was recorded in 4 to 8-month-old infants who passively viewed happy and neutral facial expressions and non faces. Although no prematurity related EEG differences were detected in 4-month-old children, 8 months olds with histories of premature birth showed less evidence for cortical specialization (based on lack of differentiation of in alpha power across facial emotion conditions and scalp regions), relative to 8 months old born full term, who showed significantly greater alpha power to happy versus neutral and non faces, especially in occipital regions (Carbajal-Valenzuela et al., 2017).

We are unaware of any work on how ERPs to facial emotion processing vary as a function of premature birth at any point in the lifespan. This study focused on risk in toddlerhood, a period of early childhood ranging between 12 months up to 36 months. There is a scarcity of ERP research in this age range, even in typically developing samples, and especially in prematurely born toddlers. Toddlerhood represents a sensitive period of social development when behavioral risk can be observed. Thus, we enrolled children born across a range of prematurity stages from 23 weeks to 36 weeks’ gestation, spanning the full range of risk extreme, very, and late preterm stages, which allowed us to examine a gradient of risk based on prematurity severity. Children ranged from 14 to 30 months of age, allowing us to examine modulating effects of age, which supports translational efforts to inform early detection and prevention efforts.

Advances in neuroimaging have allowed for greater understanding of how prematurity interrupts typical brain maturation. Even in the absence of specific brain injury (e.g. intraventricular hemorrhage, periventricular leukomalacia), prematurity influences foundational axonal growth, dendritic arborization, synaptogenesis, and pruning that dominates the early stages of brain development (Young et al., 2015; Volpe, 2009, 2019). Emerging evidence from MRI studies shows that frontal-temporal circuitries are particularly susceptible to effects of prematurity (Peterson, 2000; Bäuml et al., 2015; Hasler et al., 2020; Healy et al., 2013; Kanel et al., 2022; Kesler et al., 2008; Meng et al., 2016; Nosarti et al., 2008; Shang et al., 2019; Ball et al., 2012; Giménez et al., 2006), with effects lasting even into adulthood (Johns et al., 2019; Mossad et al., 2021; Papini et al., 2016; Rogers et al., 2017; Scheinost et al., 2016).

This study uniquely examined ERPs to dynamically changing facial expression, underutilized in ERP work, despite being more representative of how facial emotions are processed in daily life relative to static images. There is significant heterogeneity in ERP responses to face in early childhood. Leveraging dynamic facial stimuli and examining moderators like prematurity risk may help resolve current ambiguities in the field. Examining face sensitive ERPs including the N290 and P400 and Nc can shed light on developmental disruptions in perceptual and attention to faces and facial emotions. Hypotheses on effects of gestation on ERPS were motivated by work demonstrating disruptions in the development of neural circuitries that support face processing, especially in children born at very early gestational stages. Based on prior work, we hypothesized that children born at earlier gestations would show more immature ERP profiles indicated by reduced discrimination and right hemisphere lateralization (Quadrelli et al., 2019) in candidate N290, P400, and NC when children passively viewed dynamically changing positive (happy), negative (angry), and neutral facial expressions, which were presented as dynamic stimuli from neutral to from neutral to intense emotion expression. We also examined whether gestational status interacted with age to influence ERP amplitudes and latencies. We had no specific hypotheses as to whether premature birth would exert strong effects on younger or older toddlers, but explored this given that we had variability in toddler ages.

Although EEG lacks special precision, source localization techniques can be used to estimate potential neural generates of ERPs. We followed up main analyses with source localization techniques for this purpose. Using source localization methods, we hypothesized that ERPs would be localized to frontal and temporal regions associated with social cognition. More specifically, we expected that ERP activation would localize to frontal and temporal regions associated with face processing; moreover, we expected that earlier gestational status would appear as more immature (smaller in amplitude and less localized to frontal/temporal circuitries. Finally, we expected that reduced discrimination or lateralization based on ERP values would correlate with greater behavioral risk, based on parent report of internalizing and externalizing symptoms and general problem behaviors.

## 2 Methods

**Procedures.** This study included toddlers born preterm and their primary caregivers. Data for this paper were drawn from the first set of participants who completed the baseline behavioral and EEG data collection sessions as part of the larger randomized clinical trial, testing whether modifying parental responsiveness supports more neurotypical brain and behavioral in toddlers born preterm. Participants were recruited via social media, community sites, and pediatric clinics that served the needs of prematurely born infants and children. Demographic and behavioral data for this study were collected in a laboratory setting at the Children's Learning Institute in Houston, TX, as part of baseline assessment. EEG data were acquired at a research laboratory at the University of Houston. The study participants received a gift card for completing this behavioral testing session. Prior to any testing, informed consent was obtained from all parents involved in this study; children enrolled in this study were too young to provide assent for participation. The Institutional Review Board at the University of Houston and the University of Texas Health and Science Center approved all recruitment and experimental procedures for this study.

**Participants.** Eligibility criteria included being born at or before 36 weeks’ gestation and having an adjusted age between 12 and 36 months at the time of enrollment. Exclusionary criteria included 1) presence of known/suspected congenital anomalies including chromosomal or complex congenital heart disease; 2) congenital infection including TORCH (Toxoplasmosis, Rubella, Cytomegalovirus, Herpes Simplex and others), untreated maternal HIV, or maternal syphilis; 3) bilateral grade 3/4 intraventricular hemorrhage, intraparenchymal hemorrhage, hydrocephalus; 4) Cerebral palsy with Gross Motor Function Classification of III or higher (not able to sit without support); 5) blindness (child or parent); 6) deafness; 7) Current maternal drug use; 8) Maternal age under 18-years when child was born; 9) families who reside outside the catchment area (>1 h drive from the Texas Medical Center); 10) Child with contraindication for MRI.

There were a total of 48 participants who participated in the EEG assessment. Three participants were excluded from the final sample due to technical issues (n = 1), failure to meet the trial threshold (e.g., ≥20 trials per facial expression condition) after visual inspection in Brain Analyzer software (n = 1), and failure to retain a minimum of 20 epochs per facial expression condition after preprocessing procedure in HAPPE (n = 1). The final sample in this study included 45 toddlers who ranged from 15.6 to 33.67 mos (M = 21.96 mos, SD = 5.25) adjusted for prematurity. A little over half of the sample were male (53%; n = 24). Based on gestational age, 47% (n = 21) were classified as extremely preterm (22–27 weeks), 29% (n = 13) as very preterm (28–33 weeks), and 24% (n = 11) as late preterm (34–36 weeks). Parent income-to-needs ratios ranged from 1 (lowest income) to 8 (highest income), with an overall mean of 2.5 across the sample. Maternal educational attainment ranged from 1 (Primary School) to 12 (Doctorate or advanced degree), with a median of 6, corresponding to “Some college but no degree.” Length of neonatal intensive care unit (NICU) stay ranged from 0 to 30 weeks. See Table 1 for additional demographic information.

**Table 1:** Demographics.

	Preterm Group			
	Extreme Preterm (22-27 weeks) N = 21^a^	Very Preterm (28-33 weeks) N = 13^a^	Late Preterm (34-36 weeks) N = 11^a^	Full Sample N = 45^1^
Sex				
Female	9 (43%)	6 (46%)	6 (55%)	21 (47%)
Male	12 (57%)	7 (54%)	5 (45%)	24 (53%)
Race				
American Indian/Alaska Native	0 (0%)	1 (7%)	0 (0%)	1 (2%)
Asian	2 (10%)	0 (0%)	0 (0%)	2 (5%)
Black/African American	7 (33%)	3 (23%)	4 (36%)	14 (31%)
Native Hawaiian/Pacific Islander	0 (0%)	0 (0%)	1 (9%)	1 (2%)
White	5 (24%)	8 (62%)	5 (46%)	18 (40%)
NA	7 (33%)	1(8%)	1(9%)	9 (20%)
Ethnicity				
Hispanic	9 (43%)	9 (69%)	7 (64%)	25 (56%)
Not Hispanic	10 (47%)	4 (31%)	4 (36%)	18 (40%)
NA	2 (10%)	0 (0%)	0 (0%)	2 (4%)
Income	2.92 (1.00 - 8.00)	2.27 (1.00 - 8.00)	2.14 (1.00 - 5.00)	2.51 (1.00 - 8.00)
Education	6.17 (1.00 - 12.00)	5.92 (1.00 - 8.00)	5.70 (3.00 - 8.00)	5.98 (1.00 - 12.00)
NICU Time (Weeks)	15.54 (12.00 - 30.00)	9.80 (3.00 - 20.00)	1.94 (0.00 - 4.00)	10.85 (0.00 - 30.00)

The demographics tables provide demographic descriptive statistics for each prematurity group as well as the full sample.

a n (%); Mean (Min - Max).

**EEG recording.** The electroencephalogram (EEG) was recorded with a 64-electrode actiCHamp system (Brain Products GmbH) with electrodes arranged in according to a standard 10-10 system layout, with reference at FCz, and no online filtering. The sampling rate during recording was set at 500 Hz for the first ten participants; however, we increased the sampling rate to 1000 Hz for the remaining participants; all data were down sampled to 250 Hz in the preprocessing stage. Prior to the EEG recording, impedances were inspected in BrainVision Recorder software (Brain Products, Munich, Germany) and deemed acceptable if lower than 20 KΩ. Stimuli were presented using Presentation software (Neurobehavioural Systems ©), and event triggers were sent via a parallel port and recorded synchronously with the EEG data as digital event markers.

**Dynamic Face Task** The stimuli consisted of 1000-ms video clips of three different expressions (happy, angry, and neutral). These videos were specifically created for this task. Four female volunteers of different racial and ethnic backgrounds (Black, White, Hispanic, and Asian) were recruited from the Department of Psychology at the University of Houston to record videos that contain differing facial expressions. These videos were then produced in line with the protocol used in previous infant facial emotion studies (Missana et al., 2014). Face-sensitive ERPS are shown to be modulated by myriad features some of which concern bottom-up properties related to visual contrast, and others that may reflect familiarity based on gender, age and race/ethnicity (Balas et al., 2011). To mitigate effects of familiarity due to “other gender, age, and race effects” children viewed stimuli of female faces that matched their race, ethnic and cultural background (Bick et al., 2013).

**Fig. 1:**
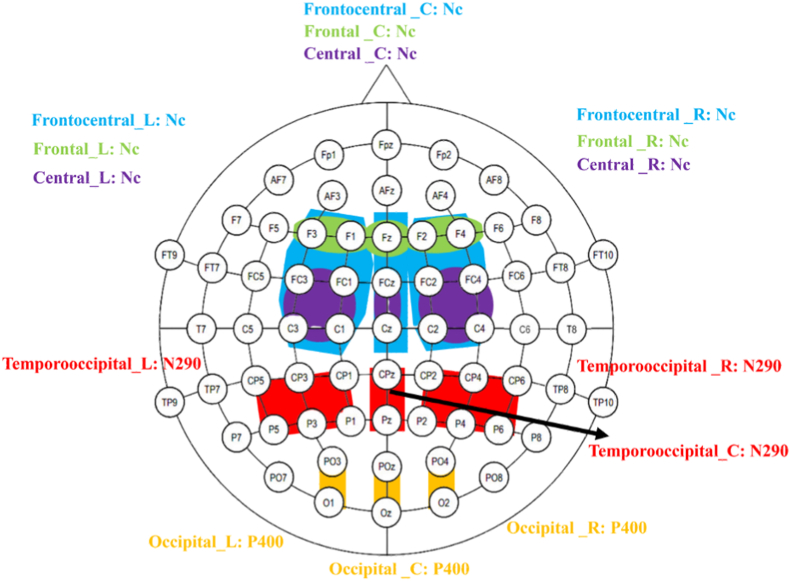
EEG channel topographical map.

**Fig. 2:**
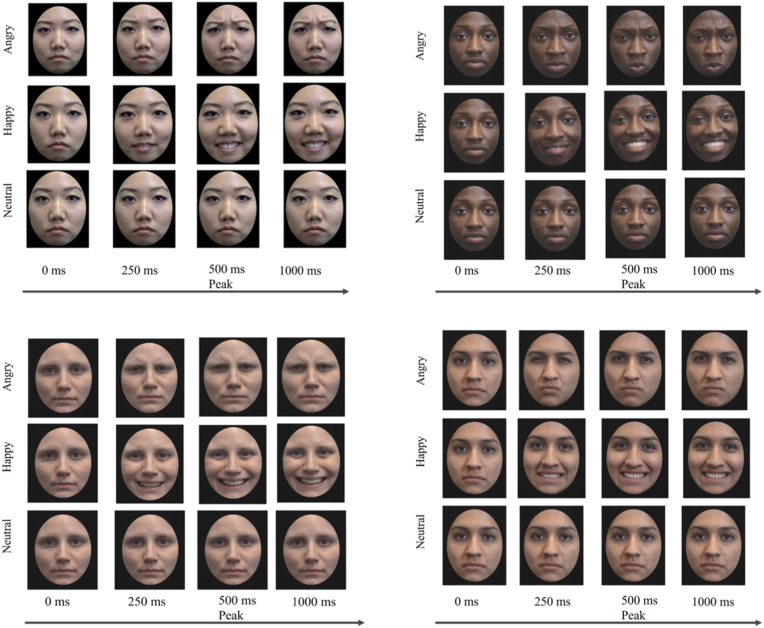
Dynamic Facial Expression Stimuli used for ERP task. Stimuli were matched to race/ethnic background of the participant.

The dynamic face task was presented using Presentation software (Neurobehavioural Systems ©). Following a fixation cross, a video clip of facial expressions (30 frames/second) appeared on the computer screen with a black background. Infants sat on their parents' lap at 70 cm away from a 21n/53 cm monitor (refresh rate 60 Hz with pixel resolution 1980 x 1080). Images/video clips were presented as a standard size (using an oval mask over each face, approximately 8.4° × 10.8°, see Fig. 2). Following prior work (Quadrelli et al., 2019), for happy and angry conditions, the video started with a neutral facial expression that gradually transitioned to peak expression (angry or happy) within the first 500 ms. The peak expression was sustained on the screen for another 500 ms. There was no inter-trial jitter because stimuli were presented manually, often not in long successive periods, given propensity for off task or distractable behavior at this age. The neutral condition consisted of a neutral face with minimal movement, continually presented across the time window. A second experimenter manually presented stimuli only when the child was looking at the computer screen. If needed, attention-grabbing videos were presented to gain the child's attention toward the computer screen. These videos contained cartoon animal figures that lasted 4 s. The second experimenter controlled the start and stop of attention-grabbers by pressing the space key. One attention-grabbing video could be shortened, or multiple videos could be shown, depending on how long it took for the child to regain attention to the screen. Once the child's attention returned to the screen, the second experimenter would press the enter key to present the next trial. Given the manual nature of the key inputs, there would be at least 1 s interval between the stop of attention grabbers and the onset of the next trial. There were 150 trials in total (i.e., 50 trials per facial expression condition). Facial emotions were presented in a pseudorandom order, ensuring that 50 trials of each condition were presented; each actor appeared in the same number of times for each emotion face. The task continued until the total number of trials were reached, or until the toddler's attention and cooperation could not be maintained anymore. See Fig. 1 for an example of the stimulus video.

**EEG processing.** Visual inspection was performed to detect no-looking behavior in Brain Analyzer software (Brain Products, Munich, Germany). Trials were marked as invalid if the toddler was not looking at the stimulus. EEG data was re-referenced to an average. The Harvard Automated Processing Pipeline for Electroencephalography, version 3.0, was used for preprocessing (Gabard-Durnam et al., 2018). Data were visually inspected at each step of the pipeline to ensure it was optimized for this sample. The HAPPE pipeline included the following steps, using a combination of EEGlab (Delorme and Makeig, 2004) and MATLAB signal processing toolbox (MathWorks, 2021) functions: line noise processing (60hz line noise reduction), resampling (down-sampling of data to 250 Hz), filtering (high-pass filter of .3Hz (consistent with infant EEG standards); low pass filter of 30Hz), bad channel detection (criteria were flatlined data of 3 s and/or correlation of less than 0.1 with nearby channels), wavelet thresholding (decomposing data into 9 levels using Coiflet-4 wavelet, and empirical bayes shrinkage estimation, to adaptively further suppresses noise while preserving EEG signal structure to the continuous data), segmentation (epochs of −100 to 1000ms relative to stimulus onset), baseline correction (−100ms to 0), segment rejection (epochs with amplitudes ± 200mv were rejected), bad channel interpolation, referencing (average reference). Data were visually inspected at each step of the pre-processing to ensure appropriateness of pre-processing parameters. All subjects had more than 50% of good channels (mean = 79.81%), meeting the threshold for inclusion in analyses. All subjects had at least 20 good epochs per facial expression condition meeting criteria for ERP analyses (happy facial expression: mean = 37.02, SD = 7.26; neutral facial expression condition: mean = 37.43, SD = 8.08; angry facial expression: mean = 37.3, SD = 7.53). Prior to ERP calculations, a baseline correction (−100 to 0) was applied to all epochs.

**ERP analyses.** ERP analysis was performed using ERPLAB (Lopez-Calderon and Luck, 2014). The mean amplitude and latency of N290, P400, and Nc were calculated. N290 was measured using time window of 190-350 ms. The P400 mean amplitude were extracted in the time window of 350-550 ms. The mean amplitude of Nc was examined in the time window of 350-550 ms. Time windows were informed by prior work (Xie et al., 2019). For seven participants, the stimuli presentation was 200 ms delayed due to a technical issue. We applied an offset correction of 200ms to subjects data, which corrected this issue, effectively shifting the trial onset trigger to the correct zero point latency. For statistical analyses, electrodes were grouped into 15 clusters of scalp regions that have been previously used to examine N290, P400, and Nc for infant face processing (Xie et al., 2019; Quadrelli et al., 2019). The “Fronto-Central” clusters were divided into left, right, and central regions and used for the Nc analysis. The left, right, and central the “Occipito-Temporal” clusters were used to analyze N290. P400 was extracted from the left, right, and central “Occipital-Inion” clusters. These regions were selected based on prior work (Xie et al., 2019) Fig. 1 specifies which channels were used for the Frontal vs Central NC, the N290, and the P400.

**Source localization methods.** Cortical source analysis was computed in MATLAB using customized scripts adapted from https://osf.io/knf9t/(Conte and Richards, 2022), using functions from Fieldtrip (Oostenveld et al., 2011) for solving the forward and inverse problem, obtaining the current source density distribution for frontal and temporal regions of interest. Realistic head models were created using MRI templates matched to the child's age, from an open Neurodevelopmental database (Gao et al., 2019), following prior work (Xie et al., 2019) MRI templates were segmented into nine tissue types (gray matter, white matter, cerebrospinal fluid, dura, skull, muscle, eyes, nasal cavity and scalp). These segmentations were used to provide an accurate description of head model compartments, as in prior work (Guy et al., 2016; Conte and Richards, 2022; Gao et al., 2019; Xie and Richards, 2017). Source dipoles restricted to gray matter and eyes were generated using hexahedral wireframe grids and a finite element level (FEM) head model (Conte and Richards, 2022). Electrode coordinates were reconstructed and registered with the MRI template in fieldtrip. The inverse solution was estimated using the exact-LORETA method (Pascual-Marqui, 2007) and applied to each person's ERP dataset. A singular value decomposition (SVD) was performed at each location of the source model to obtain a measure of the reconstructed source at each time point of each ERP epoch. SVD values across conditions were averaged within ROIs cortical regions implicated in face processing and explored in prior work (Pascual-Marqui, 2007) These included eight regions in the frontal lobe (middle and lateral orbitofrontal gyrus, the inferior, middle, and superior frontal gyrus, the gyrus rectus, the insular cortex, the cingulate gyrus), seven regions in the temporal lobe (the inferior, middle, and superior temporal gyrus, the fusiform gyrus, the superior parietal gyrus, the supramarginal gyrus, the angular gyrus) and three regions in the occipital lobe (the inferior and superior occipital gyrus, and the lingual gyrus). The source activity was calculated for each condition for each participant. Following visual inspection for each subject, the source activity was averaged across each ROI of interest and plotted to determine potential sources for the N290, P400, and NC ERPs. Group differences in source activity across ROIs were also plotted and displayed on the MRI template space for each age group. Thus, source localization analyses were conducted for descriptive and visualization purposes, and were not subjected to statistical hypothesis testing across regions of interest. Given that ages ranged from 15 to 30 months or age, individual source solutions were normalized to an MRI template that was closest in age to each participants' adjusted age at the time of the assessment. Specifically, children between 14 and 16 months adjusted age (n = 22) were normalized to a 15-month template. Children between 17 and 21 months adjusted age (n = 10) were normalized to an 18-month template. Children between 22 and 26 months adjusted age (n = 7) were normalized to a 24-month template. Children between 27 and 20 months or age (n = 6) were normalized to a 30-month template (Richards et al., 2016).

**Brief Infant-Toddler Social and Emotional Assessment (BITSEA).** The BITSEA (Briggs-Gowan, 2004) is a nationally standard, 42-item screener designed to identify behavioral problems and competencies in children ranging from 12 to 36 months of age. Parents report on child problem behaviors (such as “restless and can't sit still”, “is destructive”, or “hits, kicks and bites” and competencies (such as “follows rules”, “is affectionate”, or “points to show you something far away”) on a scale from 0 (not true/rarely), 1 (somewhat true/sometimes), or 2 or (very true/often). We used subscale scores for behavioral problems, competence, internalizing and externalizing problems in our analyses, which have shown excellent test-retest reliability (Briggs-Gowan, 2004), discriminant and predictive validity (Hungerford et al., 2015) in diverse samples.

**Data Analytic Plan.** Hypotheses regarding the effect of gestational status on ERPs of interest were using linear mixed models. The lme4 (Bates, 2010) package in R was used to fit mixed effect models. Dependent variables included mean amplitude and peak latency for the N290, P400 and NC ERPs. For all models, predictors including two within-subjects’ factors, each with three levels: Facial Expression Condition (FEC; happy, angry and neutral) and Laterality (LAT; left, midline, right) and two continuous predictors of interest: gestational age at birth and child age adjusted for prematurity. These continuous predictors were centered and scaled prior to analyses. Main effects and two- and three-way interactions were tested for all predictors. The four-way interaction between FEC, LAT, gestation and child adjusted age was not included in analyses due to power restrictions. Models were estimated by restricted maximum likelihood (REML). A random intercept for each subject was included to account for within-participant dependence across repeated observations. Residual diagnostics (e.g., homoscedasticity and normality of residuals) were inspected to confirm model adequacy. Follow up tests for main effects and interactions used Tukey-adjusted pairwise contrasts to control for multiple comparisons. To ensure sufficient power to detect main effects and interactions of interest, power analyses were conducted in R version 4.5.1. using Monte Carlo simulation of the linear mixed method used to test hypotheses. This included testing all main effects, two-way, and three-way interactions between FEC, LAT, gestation and child age adjusted for prematurity. Simulations assumed a correlation of 0.6 across repeated measures and a total sample size of 45. Effects were parameterized using Cohen's f values representing small to moderate (f = 0.20), moderate (f = 0.25) and large (f = 0.40) effect sizes. Empirical power was estimated across 1000 replicates at α = 0.05. Results indicated sufficient power to test effects even with f = 0.20. Power for main effects ranged from 0.808 to 0.940; power for two-way interactions ranged from 0.748 to 0.919 and power for three-way interactions ranged from 0.852 to 0.871. Power analyses were re-run with a median split for gestation and produced similar estimates.

In the final set of analyses, we tested functional implications of ERP components associated with gestational status and toddlers’ behavioral risk. Only ERPs that were associated with gestational status in prior models were selected for brain behavioral analyses. Given negative skew, non-parametric correlational analyses were run to test associations between ERPs of interest and internalizing, externalizing, competence, and problem behavioral on the BITSEA.

## 3 Results

**Preliminary Analyses.** Prior to analyses, all variables were inspected for outliers and non-normal distributions. All ERP dependent variables were visually inspected and confirmed with Shapiro-Wilkes tests to be normal or approximately normally distributed, meeting assumptions for repeated measures model. Outliers were defined as data points more than 3 times the standard deviation beyond the mean and were winsorized prior to analyses. For ERP data, across all conditions (angry, happy, neutral) and laterality (left, center, right), there was 1 outlier for latency and 6 outliers for mean amplitude data. For BITSEA data, there was one outlying value for the externalizing scale, and 1 outlier for the competency scale. Continuous independent variables (gestational status) or covariates of interest (age, SES) were outlier free. Family SES and child sex assigned at birth were explored as covariates in preliminary analyses. Neither was associated with dependent variables of interest and were not included in models. For follow up models examining associations between ERPs and behavioral outcomes, spearman rho correlations were used given negative skew in BITSEA subscale scores.

### 3.1 Prematurity status and ERPs

**N290 Mean Amplitude.** The linear mixed-effects model revealed a significant interaction between facial expression condition (FEC), gestational status, and child adjusted age, *F*(2, 332) = 5.561, *p* = 0.004, partial η^2^ = 0.032 (see Table 2). No other statistically significant main effects or interactions emerged. See Table 2 for full model results. To decompose the significant interaction, estimated marginal means (EMMs) were computed for children born at earlier versus later gestation (below/above the median on gestational status) and at younger versus older ages (below/above the median on age adjusted for prematurity. Tukey adjusted contrasts compared amplitudes across facial expression condition (averaging across laterality) for each subgroup. However, mean amplitudes did not significantly differ across facial emotion conditions across subgroups at the corrected level (Tukey adjusted *p* values ranged from 0.056 to 0.999).

**Table 2:** N290 mean amplitude.

N290 Mean Amplitude: Fixed Effects — Type III (Kenward–Roger)				
*Effect*	*df (num/den)*	*F*	*p*	*ηp*^*2*^*(partial)*
FEC	2, 332	1.028	0.359	0.006
LAT	2, 332	2.359	0.096	0.014
Gestation	1, 41	0.316	0.577	0.008
Adjusted age	1, 41	0.204	0.654	0.005
FEC:LAT	4, 332	0.422	0.793	0.005
FEC:Gestation	2, 332	0.588	0.556	0.004
FEC:Adjusted age	2, 332	0.728	0.483	0.004
LAT:Gestation	2, 332	0.615	0.541	0.004
LAT:Adjusted age	2, 332	0.442	0.643	0.003
Gestation:Adjusted age	1, 41	1.435	0.238	0.034
FEC:LAT:Gestation	4, 332	0.292	0.883	0.004
FEC:LAT:Adjusted age	4, 332	0.171	0.953	0.002
FEC:Gestation:Adjusted age	2, 332	5.561	0.004∗	0.032
LAT:Gestation:Adjusted age	2, 332	0.223	0.800	0.001

N290 Mean Amplitude: Random Effects			
*Component*	*Variance*	*SD*	*ICC*
(Intercept) | Subject	3.895	1.973	
Residual	7.528	2.744	
ICC (Subject)			0.341

**Fig. 3:**
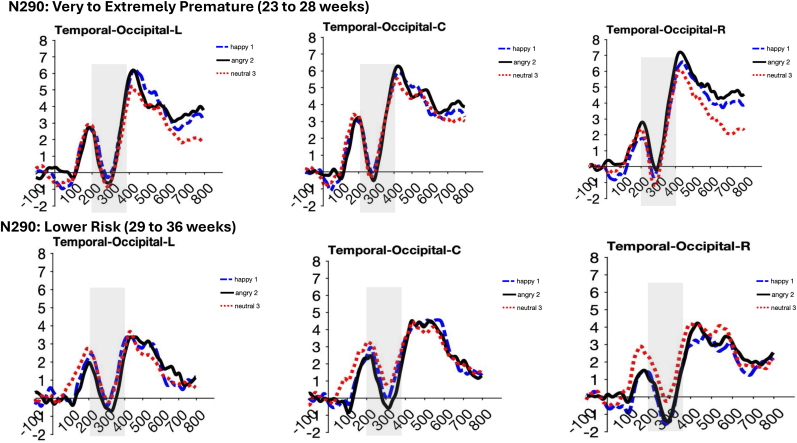
N290 ERP waveforms.

**Fig. 4:**
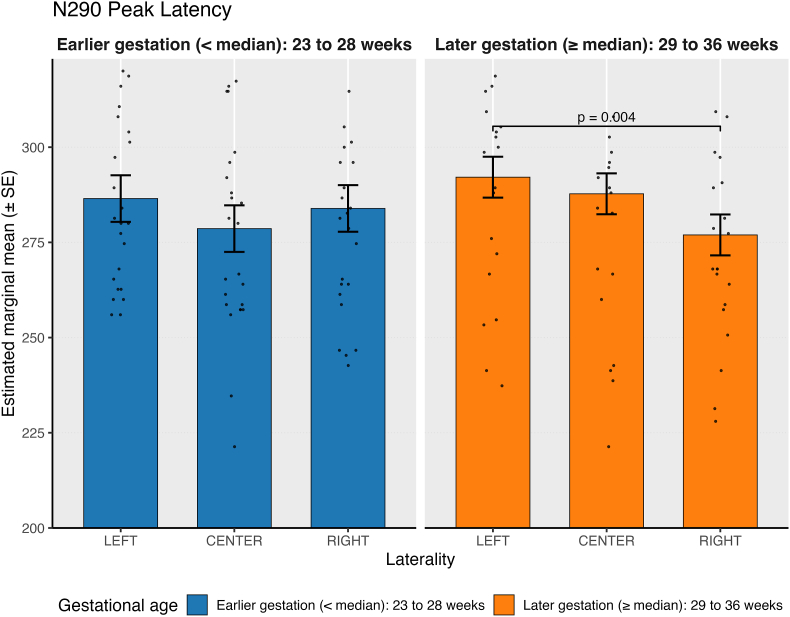
Gestation ∗ laterality interaction on N290 peak latency.

**N290 Peak Latency.** The linear mixed-effects model revealed a significant main effect of laterality, *F*(2, 332) = 5.738, *p* = 0.004, partial η^2^ = 0.033, which was qualified by a significant interaction between laterality and gestational status, *F*(2, 332) = 4.284, *p* = 0.015, partial η^2^ = 0.025. No other statistically significant main effects or interactions emerged. See Table 3 for full model results. To decompose the significant interaction, estimated marginal means were computed for children born at earlier versus later gestational stages (based on the median split) holding adjusted age at the mean for each region. Tukey-adjusted contrasts compared latencies across left hemisphere, midline, and right hemisphere amplitudes (averaging across facial expression condition) for each gestational subgroup. Significant results were indicated by confidence intervals not crossing zero. Results revealed that, for toddlers born at later gestation, N290 latencies were significantly faster over the right hemisphere relative to midline only at a trend level, *t*(332) = 2.307, *p* = 0.056, 95% CI [−0.221, 21.825] but were significantly faster between the right versus left hemisphere, *t*(332) = 3.24, *p* = 0.003, 95% CI [4.147, 26.193] regions. By contrast, for toddlers born at earlier gestation, latencies did not significantly vary across right, midline, or left hemisphere locations (all *p* values ranged from 0.302 to 0.878. Fig. 3 shows ERP waveforms differences across gestational groups split by the median. Fig. 4 shows results from post hoc tests. This is suggestive of reduced right hemispheric specialization for face processing in children born at earlier gestations, consistent with a maturational delay.

**Table 3:** N290 peak latency.

N290 Peak Latency: Fixed Effects — Type III (Kenward–Roger)				
*Effect*	*df (num/den)*	*F*	*p*	*ηp*^*2*^*(partial)*
FEC	2, 332	0.290	0.749	0.002
LAT	2, 332	5.738	0.004∗	0.033
Gestation	1, 41	0.265	0.610	0.006
Adjusted age	1, 41	2.165	0.149	0.050
FEC:LAT	4, 332	0.320	0.864	0.004
FEC:Gestation	2, 332	1.391	0.250	0.008
FEC:Adjusted age	2, 332	0.019	0.982	0.000
LAT:Gestation	2, 332	4.284	0.015∗	0.025
LAT:Adjusted age	2, 332	1.442	0.238	0.009
Gestation:Adjusted age	1, 41	0.087	0.770	0.002
FEC:LAT:Gestation	4, 332	0.936	0.443	0.011
FEC:LAT:Adjusted age	4, 332	0.487	0.745	0.006
FEC:Gestation:Adjusted age	2, 332	2.822	0.061	0.017
LAT:Gestation:Adjusted age	2, 332	0.382	0.683	0.002

N290 Mean Amplitude: Random Effects			
*Component*	*Variance*	*SD*	*ICC*
(Intercept) | Subject	426.045	20.641	
Residual	782.166	27.967	
ICC (Subject)			0.353

**Fig. 5:**
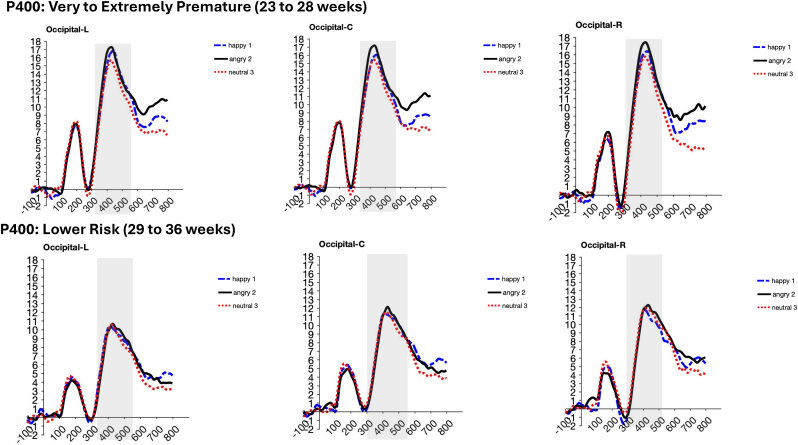
P400 ERP waveforms.

**Fig. 6:**
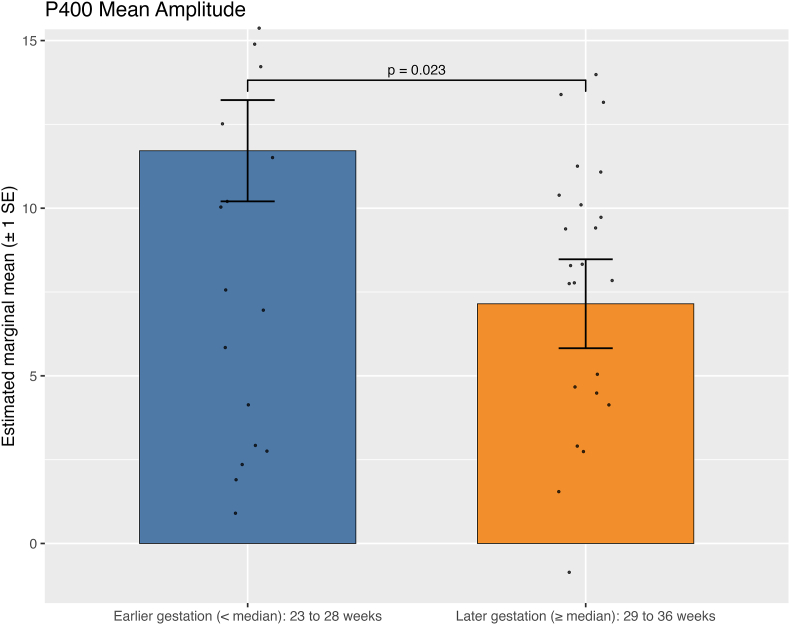
Main effect of gestation on P400 mean amplitude latency.

**P400 Mean Amplitude.** The linear mixed-effects model revealed a significant main effect of gestation, *F*(1, 41) = 5.342, *p* = 0.026, partial η^2^ = 0.115 on P400 amplitudes; no other statistically significant main effects or interactions emerged, see Table 4 for full model results. To interpret the direction of the main effect, estimated marginal means were computed for children born at earlier versus later gestational stages (based the median split) holding adjusted age at the mean. Post hoc tests (averaged across facial expression condition and laterality) revealed that children born at earlier gestation showed larger P400 amplitudes, relative to children born at later gestation, *t*(41) = −2.35, *p* = 0.023, 95% CI [−8.480, −0.649]. Fig. 5 displays P400 waveforms differences across gestational groups split at the median. Fig. 6 displays results from post hoc tests.

**Table 4:** P400 mean amplitude.

P400 Mean Amplitude: Fixed Effects — Type III (Kenward–Roger)				
*Effect*	*df (num/den)*	*F*	*p*	*ηp*^*2*^*(partial)*
FEC	2, 332	0.539	0.584	0.003
LAT	2, 332	0.813	0.445	0.005
Gestation	1, 41	5.342	0.026∗	0.115
Adjusted age	1, 41	2.264	0.140	0.052
FEC:LAT	4, 332	0.005	1.000	0.000
FEC:Gestation	2, 332	0.445	0.641	0.003
FEC:Adjusted age	2, 332	0.066	0.936	0.000
LAT:Gestation	2, 332	0.024	0.976	0.000
LAT:Adjusted age	2, 332	1.741	0.177	0.010
Gestation:Adjusted age	1, 41	0.192	0.663	0.005
FEC:LAT:Gestation	4, 332	0.105	0.981	0.001
FEC:LAT:Adjusted age	4, 332	0.102	0.982	0.001
FEC:Gestation:Adjusted age	2, 332	1.286	0.278	0.008
LAT:Gestation:Adjusted age	2, 332	1.717	0.181	0.010

P400 Mean Amplitude: Random Effects			
*Component*	*Variance*	*SD*	*ICC*
(Intercept) | Subject	39.345	6.273	
Residual	22.202	4.712	
ICC (Subject)			0.639

**Fig. 7:**
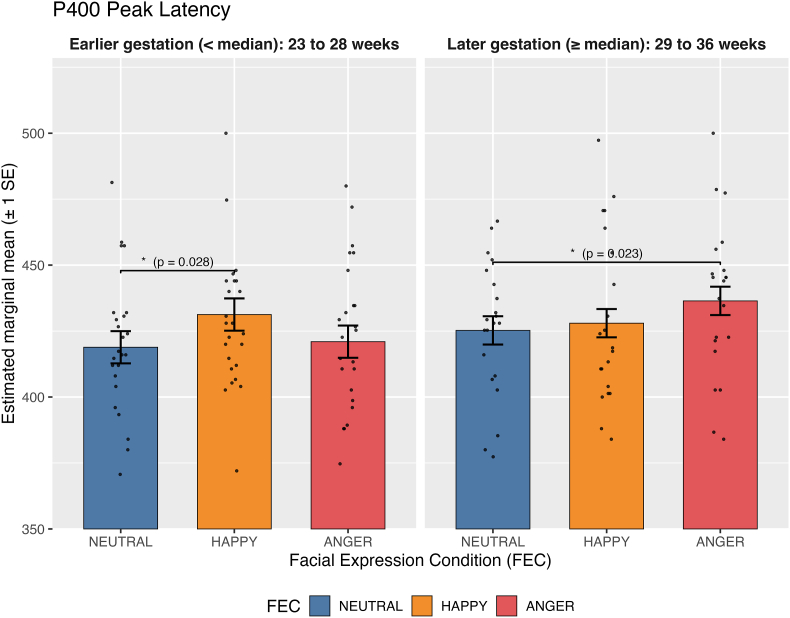
Gestation ∗ condition interaction on P400 peak amplitude latency.

**P400 Peak Latencies.** The linear mixed-effects model revealed a significant main effect of facial expression condition, *F*(2, 332) = 3.059, *p* = 0.048, partial η^2^ = 0.018, which was qualified by a significant interaction between facial expression condition and gestational status, *F*(2, 332) = 4.425, *p* = 0.013, partial η^2^ = 0.026. No other significant main effects or interactions emerged. See Table 5 for full model results. To decompose the significant interaction, estimated marginal means were computed for children born at earlier versus later gestational stages (based on a median split) holding adjusted age at the mean for each region. Tukey-adjusted contrasts compared latencies across facial expression condition (averaging across laterality) for each gestational subgroup. Results revealed that for toddlers born at later gestation, P400 latencies were relatively slower to angry versus neutral faces, *t*(332) = 2.65, *p* = 0.023, 95% CI [1.253, 21.104**]**, whereas for toddlers born at earlier gestation, P400 latencies were slower to happy versus neutral faces, *t*(332) = 2.58, *p* = 0.028, 95% CI [1.089, 23.706]. Fig. 7 shows results of post hoc tests.

**Table 5:** P400 peak latency.

P400 Peak Latency: Fixed Effects — Type III (Kenward–Roger)				
*Effect*	*df (num/den)*	*F*	*p*	*ηp*^*2*^*(partial)*
FEC	2, 332	3.059	0.048∗	0.018
LAT	2, 332	0.331	0.718	0.002
Gestation	1, 41	0.484	0.490	0.012
Adjusted age	1, 41	0.049	0.825	0.001
FEC:LAT	4, 332	1.134	0.340	0.013
FEC:Gestation	2, 332	4.425	0.013∗	0.026
FEC:Adjusted age	2, 332	0.404	0.668	0.002
LAT:Gestation	2, 332	0.562	0.570	0.003
LAT:Adjusted age	2, 332	0.356	0.701	0.002
Gestation:Adjusted age	1, 41	0.391	0.535	0.009
FEC:LAT:Gestation	4, 332	0.627	0.643	0.007
FEC:LAT:Adjusted age	4, 332	0.241	0.915	0.003
FEC:Gestation:Adjusted age	2, 332	0.424	0.655	0.003
LAT:Gestation:Adjusted age	2, 332	0.361	0.697	0.002

P400 Mean Amplitude: Random Effects			
*Component*	*Variance*	*SD*	*ICC*
(Intercept) | Subject	473.338	21.756	
Residual	634.130	25.182	
ICC (Subject)			0.427

**NC Mean Amplitude.** As a preliminary step, average NC differences were examined across frontal versus central regions. NC amplitudes over frontal regions were more negative than NC amplitudes over Central regions (*p* < 0.001). For this reason, separate models were run for frontal and central locations.

**Fig. 8:**
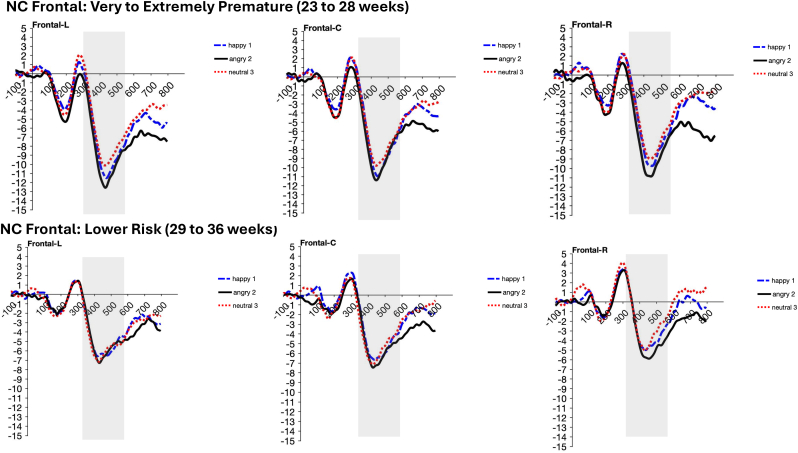
NC frontal ERP waveforms.

**Fig. 9:**
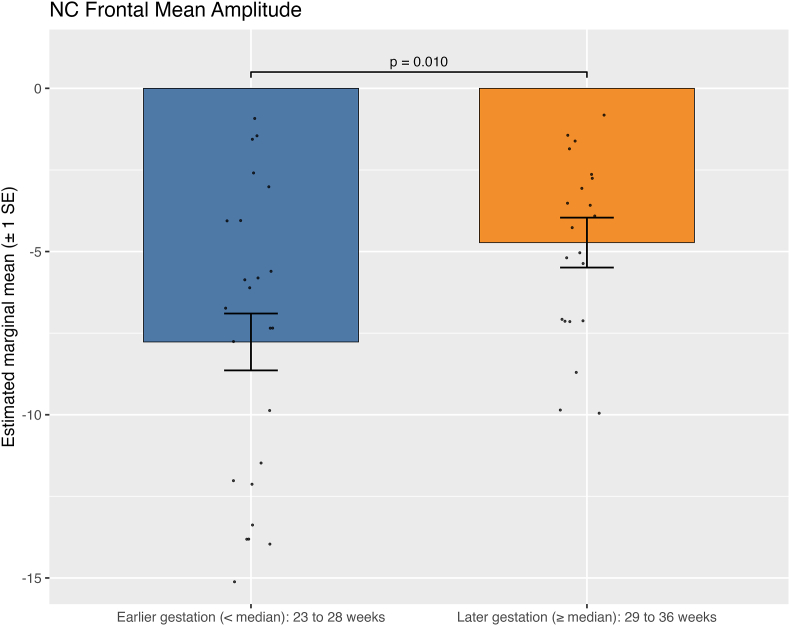
Main effect of gestation on NC frontal mean amplitude latency.

*Frontal NC.* The linear mixed-effects model revealed a significant main effect of laterality *F*(2, 332) = 6.884, *p* = 0.001, partial η^2^ = 0.040. There was also a significant main effect of gestation, *F*(1, 41) = 6.732, *p* = 0.013, partial η^2^ = 0.141. No other significant main effects or interactions emerged. See Table 6 for full model results. Post hoc tests of the main effect of laterality, compared Tukey adjusted marginal means across right hemisphere, midline, and left hemisphere regions (averaged across facial expression, and holding adjusted age and gestation at the mean). Results revealed significantly smaller (less negative) mean amplitudes in the right hemisphere relative to midline, *t*(332) = −2.56, *p* = 0.029, 95% CI [−2.463, −0.105] and left hemisphere *t*(332) = −3.61, *p* = 0.001, 95% CI [−2.985,-0.627] locations. There were no significant differences in mean amplitude between midline and left hemisphere locations, *t*(332) = −1.04, *p* = 0.551, 95% CI [−1.701, −0.627]. For Post hoc tests of the main effect of gestation, estimated marginal means were computed for children born at earlier versus later gestational stages (based on the median split) holding adjusted age at the mean. Results revealed that children born at earlier gestation showed significantly larger Frontal NC amplitudes relative to children born at later gestation, *t*(41) = 2.72, *p* = 0.010, 95% CI [0.784, 5.304]. Fig. 8 displays NC Frontal waveforms across conditions and gestational groups split at the median. Fig. 9 shows results of post hoc tests.

**Table 6:** NC frontal mean amplitude.

NC Frontal Mean Amplitude: Fixed Effects — Type III (Kenward–Roger)				
*Effect*	*df (num/den)*	*F*	*p*	*ηp*^*2*^*(partial)*
FEC	2, 332	1.839	0.161	0.011
LAT	2, 332	6.884	0.001∗	0.040
Gestation	1, 41	6.732	0.013∗	0.141
Adjusted age	1, 41	2.685	0.109	0.061
FEC:LAT	4, 332	0.215	0.930	0.003
FEC:Gestation	2, 332	0.353	0.703	0.002
FEC:Adjusted age	2, 332	0.016	0.985	0.000
LAT:Gestation	2, 332	1.062	0.347	0.006
LAT:Adjusted age	2, 332	0.723	0.486	0.004
Gestation:Adjusted age	1, 41	0.501	0.483	0.012
FEC:LAT:Gestation	4, 332	0.109	0.979	0.001
FEC:LAT:Adjusted age	4, 332	0.129	0.972	0.002
FEC:Gestation:Adjusted age	2, 332	2.225	0.110	0.013
LAT:Gestation:Adjusted age	2, 332	1.259	0.285	0.008

NC Frontal Mean Amplitude: Random Effects			
*Component*	*Variance*	*SD*	*ICC*
(Intercept) | Subject	12.048	3.471	
Residual	16.913	4.113	
ICC (Subject)			0.416

*Central NC.* The linear mixed-effects model revealed a significant main effect of child adjusted age *F*(1, 41) = 7.166, *p* = 0.011, partial η^2^ = 0.149 on NC Central mean amplitudes. This was qualified by a significant interaction between child adjusted age, facial expression condition, and gestational status *F*(2, 332) = 4.123, *p* = 0.017, partial η^2^ = 0.024. No other significant main effects or interactions emerged. See Table 8 for full model results. To decompose the significant interaction, estimated marginal means (EMMs) were computed for children born at early versus later gestation (based on a median split of gestation) and at younger versus older ages (based on a median split of age). Tukey adjusted contrasts compared amplitudes across facial expression condition (averaging across laterality) for each subgroup. Following correction for multiple comparisons, there were no significant differences in mean amplitudes across facial expression conditions that survived multiple comparisons across subgroups (Tukey adjusted *p* values ranged from 0.139 to 1.0). To interpret the main effect of age, estimated marginal means were computed for children at younger and older ages (based on a median split) holding gestation at the mean. Results revealed that older children showed significantly larger NC amplitudes relative to younger children. *t*(41) = −2.57, *p* = 0.014, 95% CI [−3.604, −0.433].

**Table 7:** NC frontal peak latency.

NC Frontal Peak Latency: Fixed Effects — Type III (Kenward–Roger)				
*Effect*	*df (num/den)*	*F*	*p*	*ηp*^*2*^*(partial)*
FEC	2, 332	4.954	0.008∗	0.029
LAT	2, 332	0.215	0.807	0.001
Gestation	1, 41	2.185	0.147	0.051
Adjusted age	1, 41	1.603	0.213	0.038
FEC:LAT	4, 332	1.016	0.399	0.012
FEC:Gestation	2, 332	2.237	0.108	0.013
FEC:Adjusted age	2, 332	0.336	0.715	0.002
LAT:Gestation	2, 332	0.416	0.660	0.002
LAT:Adjusted age	2, 332	0.535	0.586	0.003
Gestation:Adjusted age	1, 41	1.257	0.269	0.030
FEC:LAT:Gestation	4, 332	0.117	0.976	0.001
FEC:LAT:Adjusted age	4, 332	1.201	0.310	0.014
FEC:Gestation:Adjusted age	2, 332	2.549	0.080	0.015
LAT:Gestation:Adjusted age	2, 332	3.552	0.030∗	0.021

NC Frontal Mean Amplitude: Random Effects			
*Component*	*Variance*	*SD*	*ICC*
(Intercept) | Subject	650.098	25.497	
Residual	827.200	28.761	
ICC (Subject)			0.440

**Table 8:** NC central mean amplitude.

NC Central Mean Amplitude: Fixed Effects — Type III (Kenward–Roger)				
*Effect*	*df (num/den)*	*F*	*p*	*ηp*^*2*^*(partial)*
FEC	2, 332	0.223	0.800	0.001
LAT	2, 332	1.685	0.187	0.010
Gestation	1, 41	1.339	0.254	0.032
Adjusted age	1, 41	7.166	0.011∗	0.149
FEC:LAT	4, 332	0.250	0.910	0.003
FEC:Gestation	2, 332	0.251	0.778	0.002
FEC:Adjusted age	2, 332	0.933	0.395	0.006
LAT:Gestation	2, 332	0.988	0.374	0.006
LAT:Adjusted age	2, 332	0.974	0.379	0.006
Gestation:Adjusted age	1, 41	0.235	0.631	0.006
FEC:LAT:Gestation	4, 332	0.197	0.940	0.002
FEC:LAT:Adjusted age	4, 332	0.499	0.737	0.006
FEC:Gestation:Adjusted age	2, 332	4.123	0.017∗	0.024
LAT:Gestation:Adjusted age	2, 332	1.738	0.177	0.010

NC Central Mean Amplitude: Random Effects			
*Component*	*Variance*	*SD*	*ICC*
(Intercept) | Subject	11.377	3.373	
Residual	9.659	3.108	
ICC (Subject)			0.541

**NC Peak Latency.** As with mean amplitudes, separate models were run for frontal and central locations.

*Frontal NC.* The linear mixed-effects model revealed a significant main effect of facial expression condition *F*(2, 332) = 4.954, *p* = 0.008, partial η^2^ = 0.029. There was also a significant interaction between laterality, gestation, and child adjusted age, *F*(2,332) = 3.552, *p* = 0.030, partial η^2^ = 0.021. No other significant main effects or interactions emerged. See Table 7 for full model results. Post hoc tests of the main effect of facial expression condition compared Tukey adjusted marginal means across angry, happy and neutral facial expression conditions (averaged across laterality, and holding gestation and age adjusted for prematurity at the mean). Results revealed that latencies to happy faces were significantly slower than latencies to angry *t*(332) = −3.44, *p* = 0.002, 95% CI [−22.056,-4.132] and neutral *t*(332) = 2.41, *p* = 0.043, 95% CI [0.215, 18.139] faces. There were no significant differences in latencies between angry and neutral faces, *t*(332) = −1.03, *p* = 0.559, 95% CI [−12.879, 5.045]. To decompose the significant interaction between laterality, gestation, and child adjusted age, estimated marginal means (EMMs) were computed for children born at early versus later gestation (based on a median split of gestation) and at younger versus older ages (based on a median split for adjusted for prematurity). Tukey adjusted contrasts compared latencies across laterality (averaging across facial expression condition) for each subgroup. Following correction for multiple comparisons, there were no significant differences in mean latencies across facial expression conditions that survived multiple comparisons across subgroups (Tukey adjusted *p* values ranged from 0.195 to 0.979).

*Central NC.* The linear mixed-effects model revealed a significant main effect of facial expression condition *F*(2, 332) = 4.842, *p* = 0.008, partial η^2^ = 0.028, which was qualified by a significant two-way interaction between facial expression condition and child age adjusted for prematurity, *F*(2, 332) = 4.785, *p* = 0.009, partial η^2^ = 0.028. There was also a significant interaction between facial expression condition, laterality and gestational status. No other significant main effects or interactions emerged. See Table 9 for full model results. To decompose the significant interaction between facial expression and child adjusted age, estimated marginal means (EMMs) were computed for younger versus older children (based on a median split). Tukey adjusted contrasts compared latencies across facial expression condition (averaging across laterality) for each subgroup. Results revealed that younger children showed significantly slower latencies to happy faces versus angry *t*(332) = −4.45, *p* < 0.001, 95% CI [−39.676, −12.216] and neutral *t*(332) = 2.82, *p* = 0.014, 95% CI [2.731, 30.192] faces. There were no significant differences in latencies between angry and neutral faces, *t*(332) = −1.03, *p* = 0.559, 95% CI [−12.879, 5.045]. Older children showed no significant differences in latencies across facial expression conditions (p values ranged from 0.645 to 0.725).

**Table 9:** NC central peak latency.

NC Central Peak Latency: Fixed Effects — Type III (Kenward–Roger)				
*Effect*	*df (num/den)*	*F*	*p*	*ηp*^*2*^*(partial)*
FEC	2, 332	4.842	0.008∗	0.028
LAT	2, 332	0.377	0.687	0.002
Gestation	1, 41	0.007	0.932	0.000
Adjusted age	1, 41	0.972	0.330	0.023
FEC:LAT	4, 332	0.556	0.694	0.007
FEC:Gestation	2, 332	2.891	0.057	0.017
FEC:Adjusted age	2, 332	4.785	0.009∗	0.028
LAT:Gestation	2, 332	0.478	0.620	0.003
LAT:Adjusted age	2, 332	0.359	0.699	0.002
Gestation:Adjusted age	1, 41	1.775	0.190	0.041
FEC:LAT:Gestation	4, 332	3.445	0.009∗	0.040
FEC:LAT:Adjusted age	4, 332	0.796	0.528	0.010
FEC:Gestation:Adjusted age	2, 332	1.583	0.207	0.009
LAT:Gestation:Adjusted age	2, 332	0.391	0.677	0.002

NC Central Mean Amplitude: Random Effects			
*Component*	*Variance*	*SD*	*ICC*
(Intercept) | Subject	687.583	26.222	
Residual	1292.877	35.957	
ICC (Subject)			0.347

**Fig. 10:**
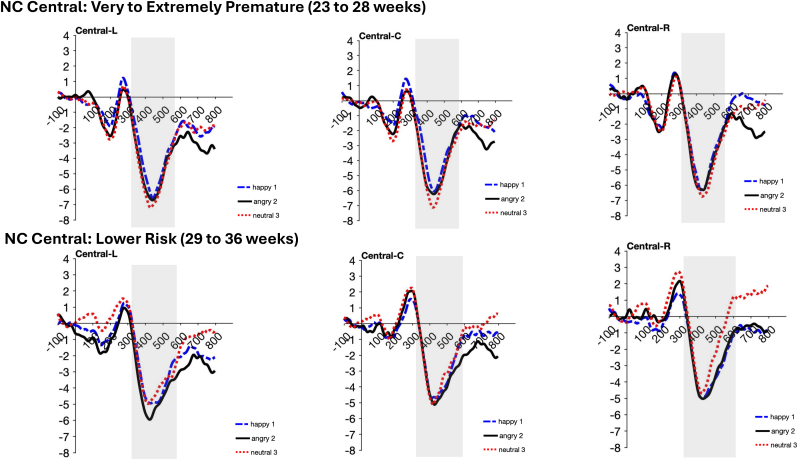
NC central ERP waveforms.

**Fig. 11:**
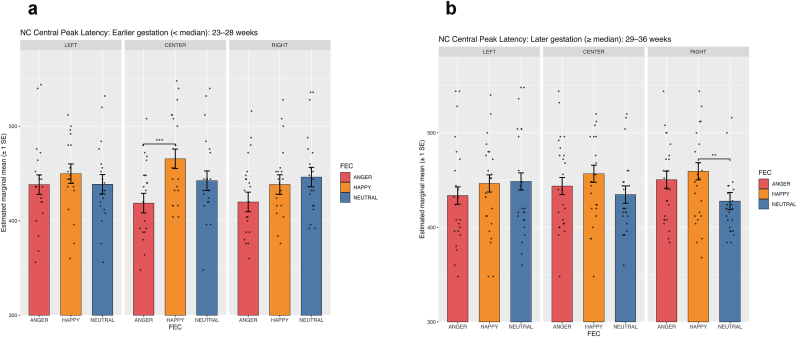
A and B. Gestation ∗ Condition ∗ Laterality on NC Frontal Mean Amplitude Latency: Panel A shows results for babies born extremely to very preterm; Panel B shows results for babies born late preterm.

To decompose the significant interaction between facial expression condition, laterality, and gestational status, estimated marginal means (EMMs) were computed for children born at earlier versus later gestations (based on a median split). Tukey adjusted contrasts compared latencies across facial expression condition and laterality for each subgroup. Results revealed children born at later gestation shows differences in latencies across the right hemisphere: specifically, latencies were faster for neutral relative to happy faces *t*(332) = 3.05, *p* = 0.007, 95% CI [7.217, 55.751]. In contrast, children born at earlier gestations showed significant different latencies in midline regions only; specifically, latencies to angry faces were faster to happy faces, *t*(332) = -4.02, *p* < 0.001, 95% CI [−74.234,-19.391], with latencies to neutral faces falling midway but not significantly differing from happy (p = 0.117) or angry (p = 0.106) faces. Fig. 10 displays NC Central waveforms across conditions and gestational groups split at the median. Fig. 11A and B shows results of post hoc tests.

**Fig. 12:**
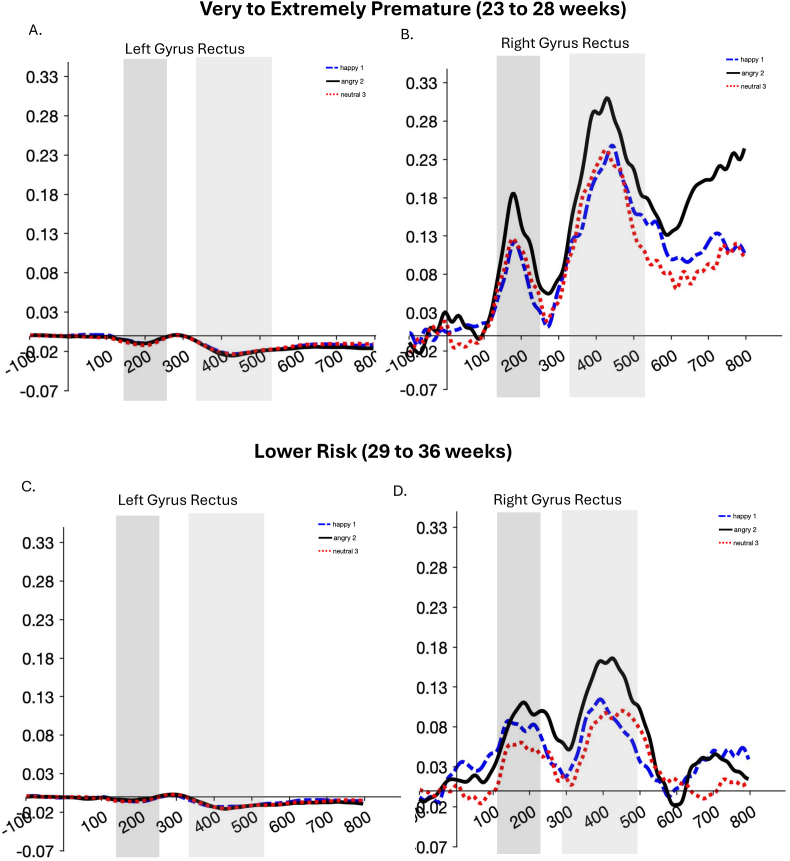
A and B. Source Localization Results: N290 is localized to the Medial Frontal Region (Gyrus Rectus). Panel A shows results for babies born extremely to very preterm; Panel B shows results for lower risk babies born late preterm.

**Fig. 13:**
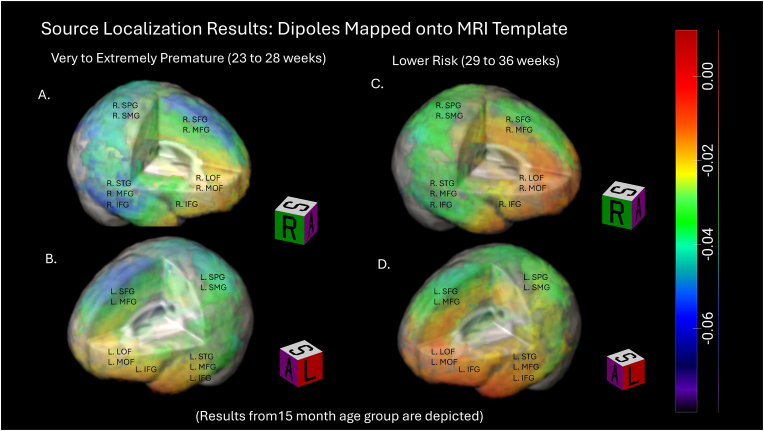
A through D. Source Localization Results: NC is localized to frontal and temporal regions. Panels A and B show results for babies born extremely to very preterm (A: right hemisphere; B: left hemisphere); Panel C and D shows results for lower risk babies born late preterm (C: left hemisphere; D: right hemisphere).

**Source localization Results.** We conducted source analyses to investigate the neural generators of ERP components examined above. Average ERP waveforms for each source location of interest were computed and plotted for visual inspection. Visual inspection of source activation waveforms indicated the right gyrus rectus in the mid-frontal lobe as a generator of both the N290 and P400 ERP components, see Fig. 12. The NC component was apparent across regions in frontal, temporal, parietal, and occipital lobes, see Figs. S1–S9. Dipole-related source activity at the peak NC latency (360 ms) was plotted on the MRI template at the whole-brain level. Fig. 13 visually displays source localization differences across right and left hemispheres and as related to prematurity status. Source activation patterns in toddlers born very to extremely prematurely (GA between 23 and 28) are plotted in Fig. 12A and B. Source activation patterns in toddlers born closer to term (GA between 29 and 36 weeks) are plotted in Fig. 13. Results of these plots indicate that frontal and temporal regions were stronger generators of the NC component than parietal and occipital regions Source localization plots also reveal group differences in NC amplitudes that align with the ERP findings. Specifically, earlier gestational age is associated with more negative (i.e., stronger) NC amplitudes.

**Fig. 14:**
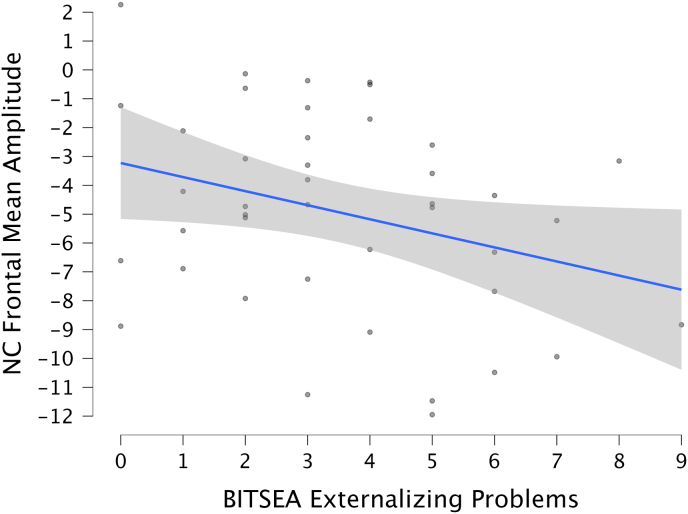
Greater NC Frontal Mean Amplitudes associated with increased risk for parent-reported externalizing problems.

**Fig. 15:**
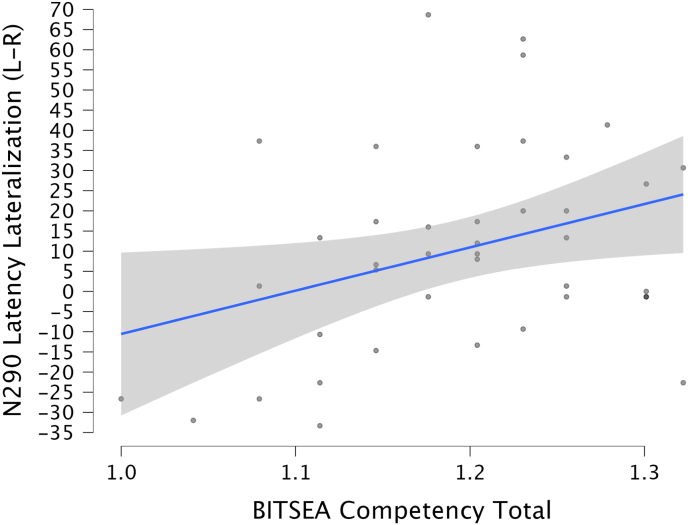
Greater N290 right vs left lateralization is associated with higher parent reported competence.

**Functional Implications.** Informed by results from prior models, N290, P400, and NC and amplitudes were examined in association with parent-reported problem behaviors, competence, internalizing and externalizing behaviors on the BITSEA (final n = 44 for these analyses). Hypotheses regarding the associations between ERPs of interest and behavioral risk variables were tested in correlational analyses. To limit the number of analyses, we only tested ERPs that associated with gestation in prior analysis. Average P400 and frontal NC amplitudes (reflecting the main effects of gestation) and central NC latency differences across affective and neutral images (reflecting the gestation by condition interaction) and N290 latency differences across right minus left regions (reflecting gestation by laterality interaction) were examined in associations with behavioral variables. More negative (larger) frontal NC amplitudes over frontal regions (which we found more characteristic of children born at more extreme prematurity) significantly correlated with greater risk for externalizing problems, *rho* = −0.299, *p =* 0.046 (small to medium effect size), see Fig. 14. Faster N290 latencies over right versus the left hemispheres (characteristic of children born closer to term, indicated by a more positive difference score) are associated with higher competency scores (*rho* = 0.252, *p* = 0.053; small effect size), see Fig. 15. These effects should be interpreted with caution, given the small sample size.

## 4 Discussion

This study expanded the understanding of how preterm birth influences the processing of facial emotions by measuring variation in ERPs associated with toddlers’ passively viewing dynamic facial expressions. Drawing on existing literature, we expected that more extreme prematurity would be associated with biases in processing expressions negative versus neutral or happy faces. We also expected that these biases would be associated with increased socioemotional risk at the behavioral level. Given that prematurity disrupts normative brain maturation, we expected it would interfere with the development of network specialization and right hemispheric dominance for face processing, which typically emerges in early childhood. To gain insight into neural networks or locations that might generate ERP activation profiles, we applied source localization methods and probed candidate regions known to support face processing and emotional regulation. Finally, we explored the functional implications of ERP variability for toddler social-emotional adjustment.

In terms of the N290, gestational status may be linked to latency differences but not amplitudes. Specifically, toddlers born at early gestational stages showed no evidence for right hemispheric specialization based on latency differences across facial emotion conditions. In contrast, toddlers born closer to term showed relatively faster latencies in right hemisphere locations relative to left hemisphere locations. Faster latencies may indicate greater specialization for face processing in right hemisphere locations, which is an established characteristic of neurotypical brain development and lateralization (Adibpour et al., 2017). This may provide support for the notion that prematurity disrupts normative brain maturation and specialization of fronto-temporal circuitries that support social cognition. It is surprising that N290s latencies did not differ across emotion facial expression condition, however, other work by Xie et al. (2019) (Xie et al., 2019), for example, suggests N290 differentiation across facial emotion conditions tends to decrease from infancy to toddlerhood.

Toddlers born at earlier gestational stages showed larger P400 mean amplitudes relative to children born closer to term, although amplitude effects did not differ based on facial emotion condition or across hemispheres. P400 latencies may reflect more sensitive to facial emotion condition such that children born closer to term shows slower latencies to angry versus neutral faces; latencies to happy faces falling in between angry and neutral expressions; children born at earlier gestation showed significantly slower latencies to happy versus neutral face with responses to angry falling in the midpoint. This could suggest that infants born at earlier gestation show less lateralized specialization when processing positive emotions, in contrast to more lateralization in children born closer to term.

In terms of NC components, toddlers born at earlier gestational stages showed larger (more negative) frontal NC amplitudes, than children born closer to term. Although unexpected, the direction of effects is consistent with findings indicating larger P400 amplitudes in children born at greater gestational risk. Larger NCs may indicate greater allocation of attentional resources when processing facial emotional input. Other studies of infants and toddlers show a waning of NC components to familiar face stimuli as they grow older, potentially reflecting increased expertise and reduced attention resources allocated to the facial stimuli, which leave the attention system more flexible to process new information in the social environment (Carver et al., 2003). Therefore, larger NC (and P400 responses) may reflect a more neurodevelopmentally immature social cognitive profile, likely characteristic of children born at early gestational stages.

Similar to N290 and P400 effects, NC latencies, especially over central regions were associated with more sensitivity to facial emotional conditions and modulated by prematurity status. Specifically, children born closer to term showed greater differential latencies in right hemisphere locations; NCs were slowest to happy faces and fastest to neutral faces; latencies to angry faces fill midway between the other two categories. However, latency differences across conditions were not shown in right hemisphere locations in children born at earlier stages of gestation. Lack of right hemispheric differentiation coincides with N290 and P400 latency findings also indicating reduced right hemispheric specialization secondary to earlier gestational status. This also highlights the value of ERP metrics for elucidating perhaps more subtle differences in neural processing of varying affective and social input that may not be evident at behavioral levels.

Given the substantive range in child developmental stage in this sample, we also considered how effects of gestation on ERP outcomes may differ as a function of child age. In general, gestational status was a stronger influence on ERP components than child age adjusted for prematurity, with the exception of NC amplitudes over central locations. Consistent with prior work (Carver et al., 2003; Webb et al., 2005), NCs amplitudes were larger in younger versus older children versus older children, especially over central regions. If NCs are reflective of a “younger” neurodevelopmental profile, we can interpret the larger NCs in children born at earlier gestational stages as a further sign of developmental immaturity of the organization of the social brain. Significant interactions between child age and gestational status emerged but were small in size and post hoc analyses were inconclusive. Replication that includes larger samples may be necessary to determine the robustness of these effects.

Cortical source reconstruction suggested that mid frontal regions subserved the N290/P400 component, which is consistent with some source localization work (Guy et al., 2016) and broader understanding that mid frontal regions support some forms of emotion processing (Bechara, 2000). NC amplitudes were most strongly localized to frontal and temporal regions, which is consistent with prior work on neural substrates of face processing (Conte and Richards, 2022). We used a standard MRI template that was closest to each subjects’ adjusted age, similar to prior work, but future work should consider additional approaches that better model the premature brain. Brain behavioral analyses revealed ERP alterations associated with prematurity have functional implications. More negative frontal NCs and reduced right hemisphere lateralization of the N290 were associated with higher behavioral risk, based on parent report on the BITSEA. While promising, these findings should be interpreted cautiously. There is a need to increase samples sizes in studies of brain behavioral correlations to ensure that findings are trustworthy and replicable. This is a key future direction and next step for our team. Given the cross-sectional design, mediational models were not conducted, so causation or directionality cannot be inferred. Continued efforts that leverage longitudinal prospective design are an important next step.

This study was designed to take a multi-modal, multimethod approach to identify neural substrates of socioemotional risk in toddlers born preterm. Despite design strengths, limitations should be considered for interpretation of findings and to guide ongoing work. First, the ERP task did not include a non-face condition. This was an intentional design choice to limit task length and support feasibility but also means a tradeoff stronger internal validity. Second, we used conventional methods for determining ERP mean amplitudes and latencies, which allowed us to draw from past literature using similar methods. According to best practices in ERP work (Luck and Gaspelin, 2017), our windows of interest for each component were established on an *a-priori* basis to limit risk for type I errors. That said, our future work will leverage more data driven mass-univariate approaches to test robustness of effects revealed in this study. We assume that ERP findings reflect alterations in neural substrates that support information processing of facial emotional input. However, we cannot rule out the fact that additional morphological differences that vary as a function of gestation (such as related to skull thickness or tissue density, which may influence signal strength) also contribute to ERP differences.

Other limitations of the task design include a lack of inclusion of non-face stimuli, to serve as controls. Exposure to negatively valenced faces was limited to angry expressions; examining associations to fearful expression has shown strong value in other work (Leppänen et al., 2007; Hoehl and Striano, 2008; Yrttiaho et al., 2014; Nelson and De, 1996), and should be considered in future studies. Stimuli were designed to have minimal differences in contract or other perceptual features that may contribute to ERP amplitudes, although we did apply smoothing features to our images. Given challenges with collecting ERP data with young children, trial counts for ERP conditions are typically smaller than those of older youth and adults. Issues related to signal to noise ratio and reliability of ERPs are always considerations in infant neuroimaging studies. In terms of strengths, this is one of the first studies to examine how prematurity or other perinatal risk factors influence these processes. We leveraged multimodal imaging techniques (EEG, MRI) thereby providing both temporal and spatial information resulting in a more comprehensive understanding of how the emotional and face processing systems operate in this vulnerable understudied sample. Our aim to connect ERPs with behavioral functioning also offers important implications for progress in prevention and intervention.

In summary, this study presents evidence that children with the earliest gestational ages may display altered neural correlates of face processing while viewing dynamic facial expressions. Supporting hypotheses, early gestational status was associated with altered ERP right hemispheric specialization for face processing, reflected in latency differences in N290, P400, and NC components. Earlier gestational status associated with atypically larger, more developmentally immature, P400 and NC amplitudes to face stimuli, although effects did not vary across emotion condition or hemisphere. Finally, we show preliminary connections between ERP components and domains of behavioral risk associated with prematurity. These findings enhance understanding of mechanisms that increase social cognitive or emotional risk in prematurely born children elucidating new targets that can be leveraged for early prevention and intervention.

## CRediT authorship contribution statement

**Johanna Bick:** Writing – review & editing, Writing – original draft, Visualization, Supervision, Methodology, Investigation, Funding acquisition, Formal analysis, Data curation, Conceptualization. **Xinge Li:** Writing – review & editing, Methodology. **Haley Laughlin:** Writing – review & editing, Visualization, Formal analysis, Data curation. **Andrea Jimenez Ortiz:** Writing – review & editing, Project administration, Data curation. **Anna Galvan:** Writing – review & editing, Project administration, Data curation. **Kelly Vaughn:** Writing – review & editing, Investigation. **Cara Price:** Supervision, Project administration, Methodology, Data curation. **Susan Landry:** Supervision, Investigation, Funding acquisition, Conceptualization. **Dana DeMaster:** Writing – review & editing, Supervision, Project administration, Investigation, Funding acquisition, Conceptualization.

## Author note

This paper was supported by funding from NIH R01HD100560 to Bick, DeMaster, and Landry.

## Declaration of competing interest

The authors declare that they have no known competing financial interests or personal relationships that could have appeared to influence the work reported in this paper.

## Data Availability

The data that support findings of the current study are available upon request from the corresponding author. The data are not publicly available due to privacy or ethical restrictions.
