# Supplementary material for: Atypical neural responses to dynamically changing facial expressions correlate with behavioral risk in toddlers born preterm: Evidence from an ERP study
Source: Neuroimage Rep. 2026 Mar 22;6(2):100333. doi: 10.1016/j.ynirp.2026.100333 (PMC13282526; doi:10.1016/j.ynirp.2026.100333)
Supplement: Multimedia component 1 [file mmc1.pdf]

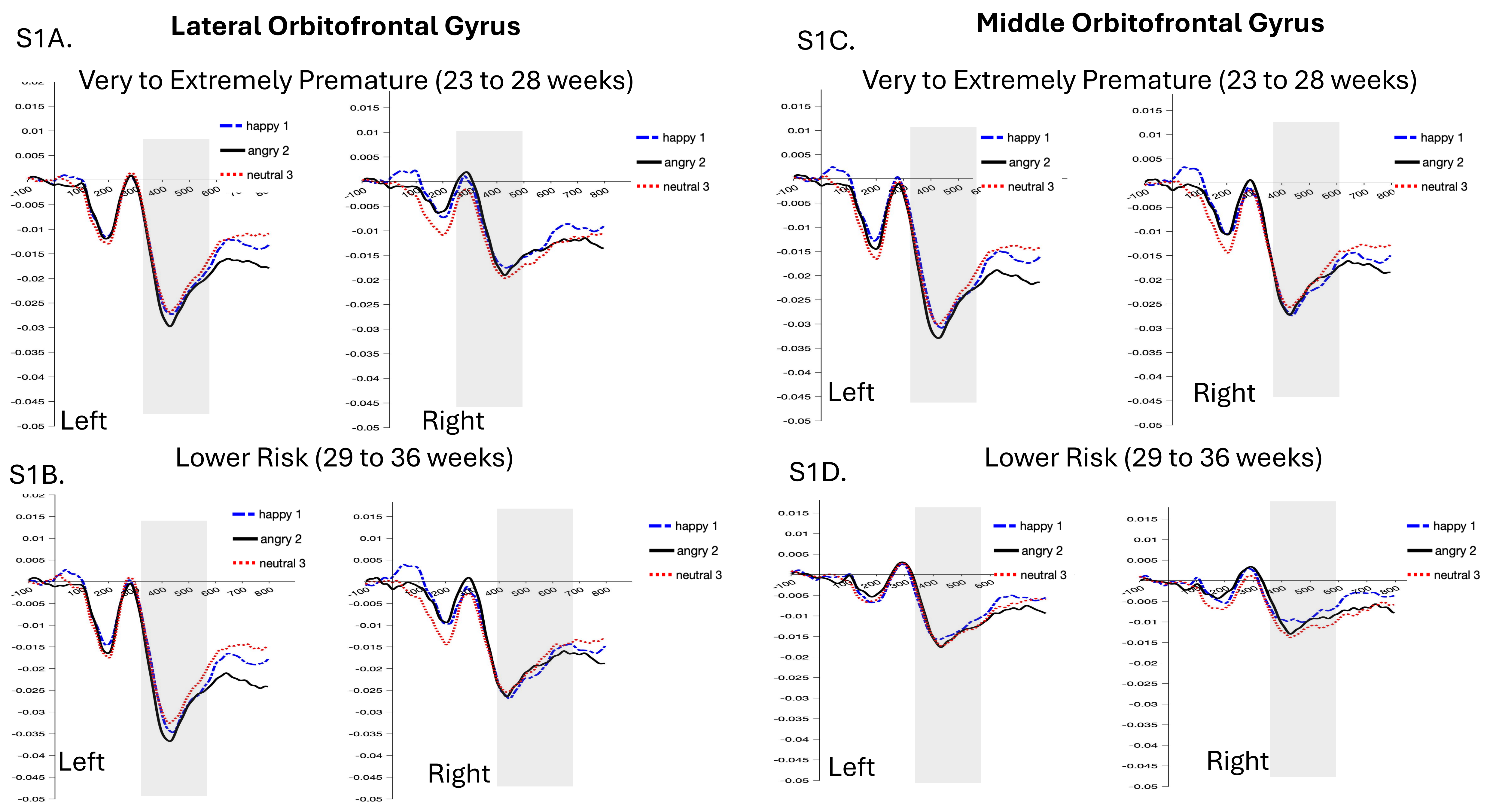

S1A.
Lateral Orbitofrontal Gyrus
Very to Extremely Premature (23 to 28 weeks)
happy 1
angry 2
neutral 3
Left
Right
S1B.
Lower Risk (29 to 36 weeks)
happy 1
angry 2
neutral 3
Left
Right
S1C.
Middle Orbitofrontal Gyrus
Very to Extremely Premature (23 to 28 weeks)
happy 1
angry 2
neutral 3
Left
Right
S1D.
Lower Risk (29 to 36 weeks)
happy 1
angry 2
neutral 3
Left
Right

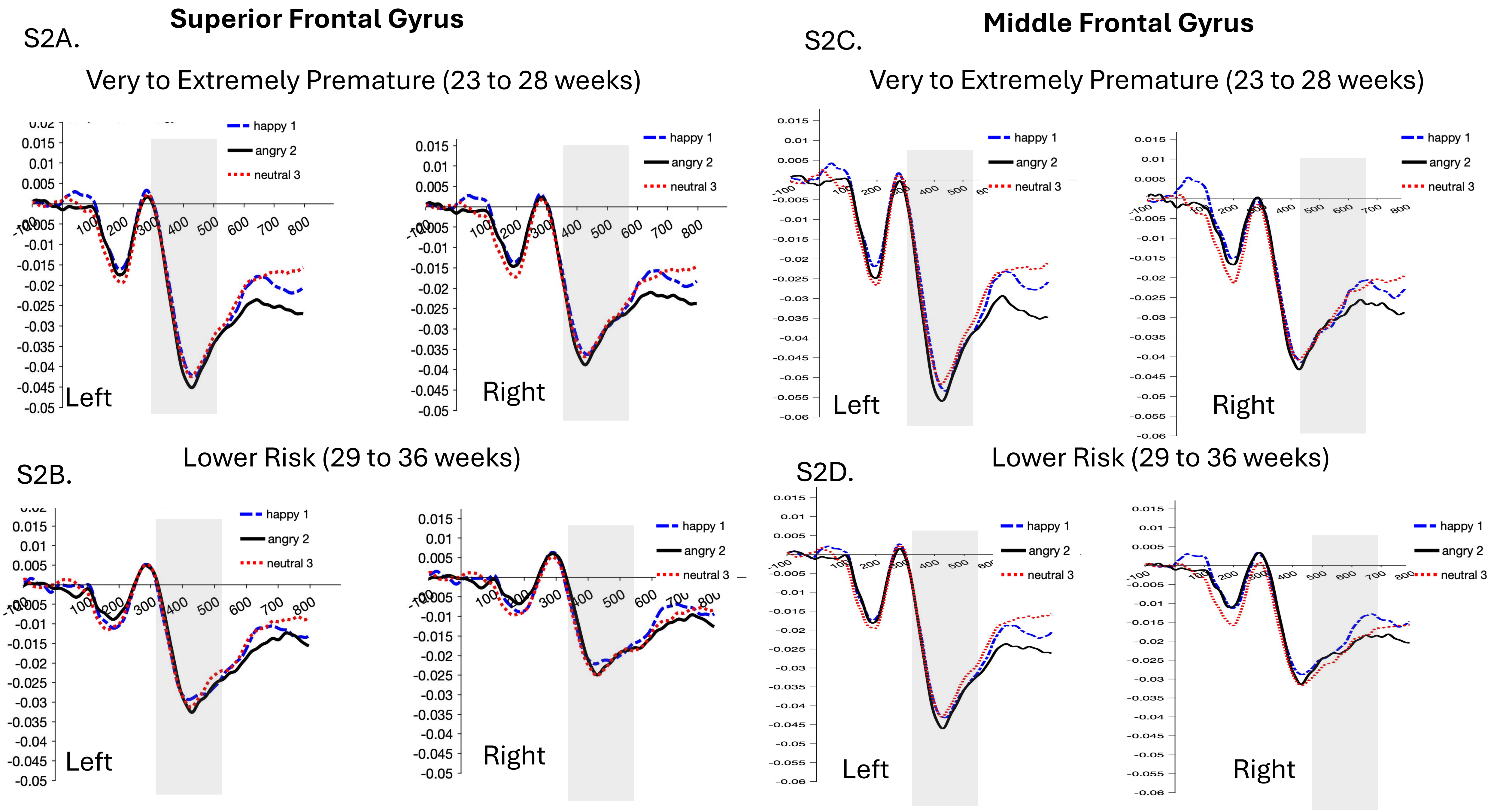
Superior Frontal Gyrus
S2A.
Very to Extremely Premature (23 to 28 weeks)
happy 1
angry 2
neutral 3
Left
Right
S2B.
Lower Risk (29 to 36 weeks)
Left
Right
Middle Frontal Gyrus
S2C.
Very to Extremely Premature (23 to 28 weeks)
Left
Right
S2D.
Lower Risk (29 to 36 weeks)
Left
Right

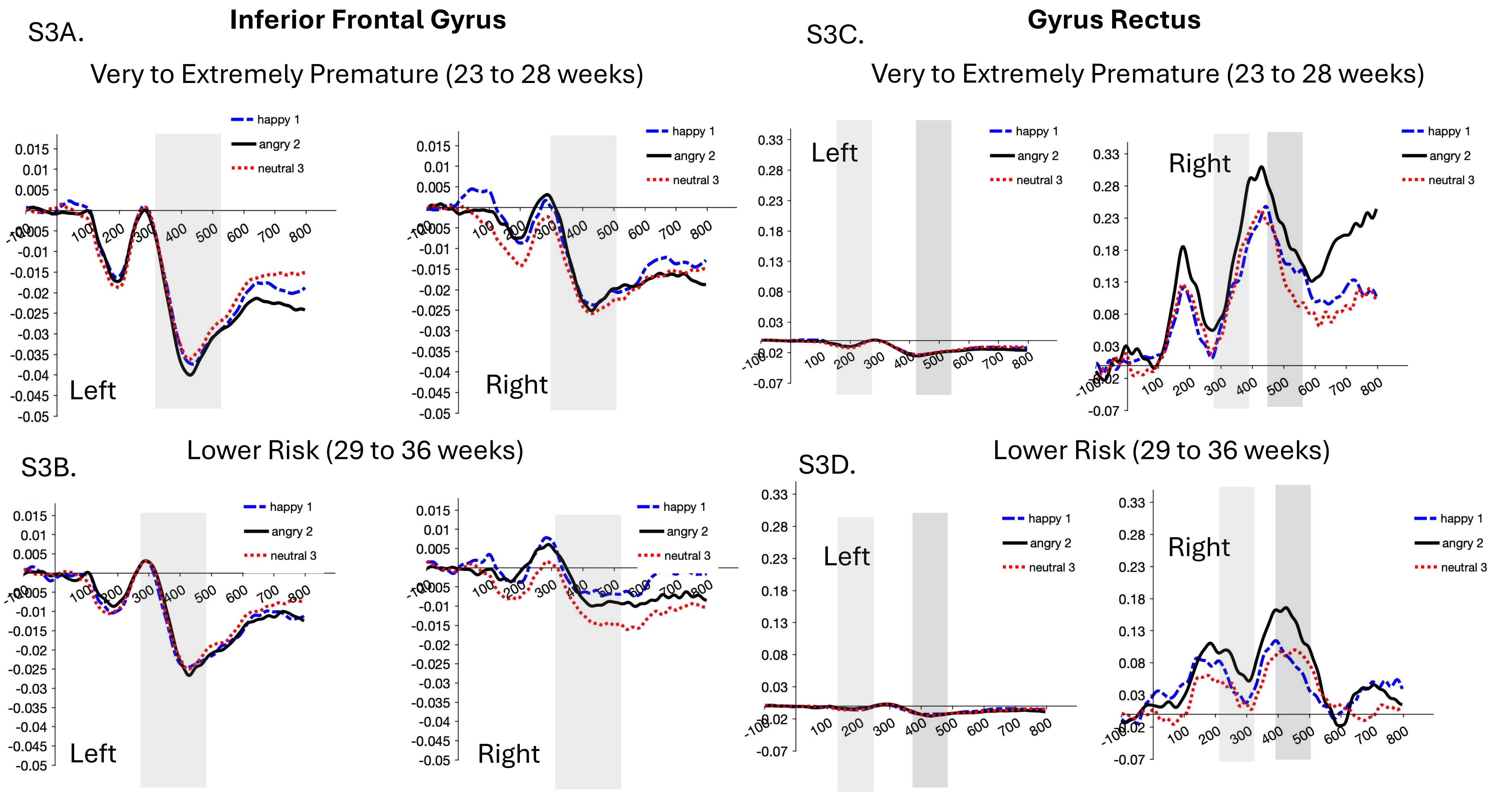
S3A.
Inferior Frontal Gyrus
Very to Extremely Premature (23 to 28 weeks)
happy 1
angry 2
neutral 3
Left
Right
S3B.
Lower Risk (29 to 36 weeks)
Left
Right
S3C.
Gyrus Rectus
Very to Extremely Premature (23 to 28 weeks)
Left
Right
S3D.
Lower Risk (29 to 36 weeks)
Left
Right
-100
100
200
300
400
500
600
700
800

Cingulate Gyrus

S4A.

Very to Extremely Premature (23 to 28 weeks)

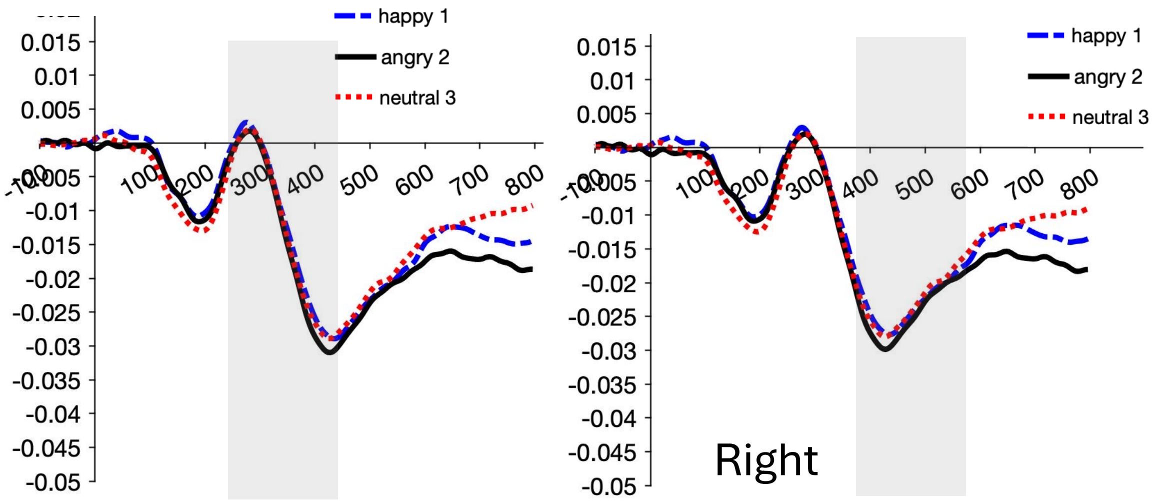

Left

S4B.

Lower Risk (29 to 36 weeks)

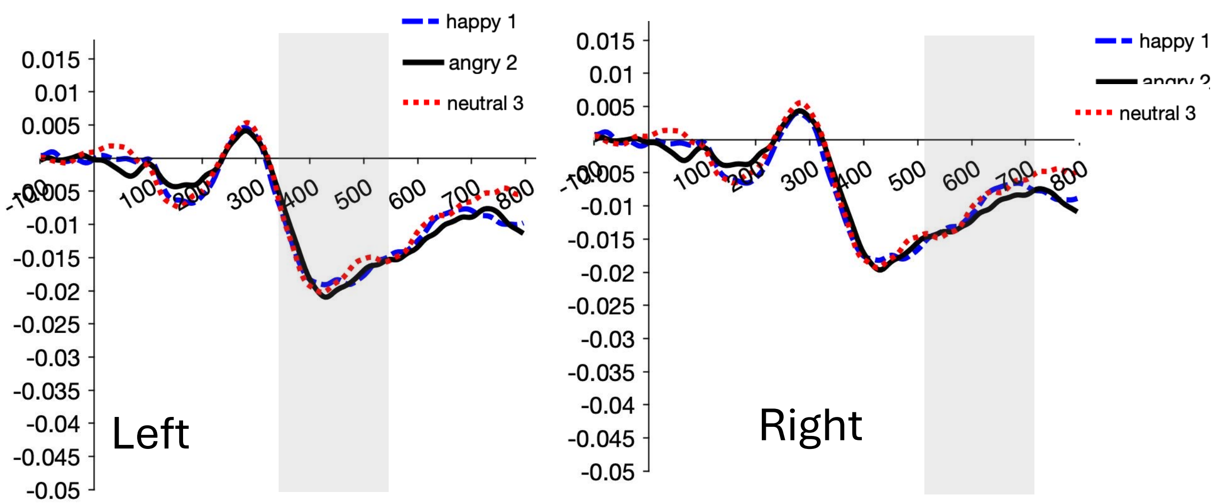

Insular Cortex

S4C.

Very to Extremely Premature (23 to 28 weeks)

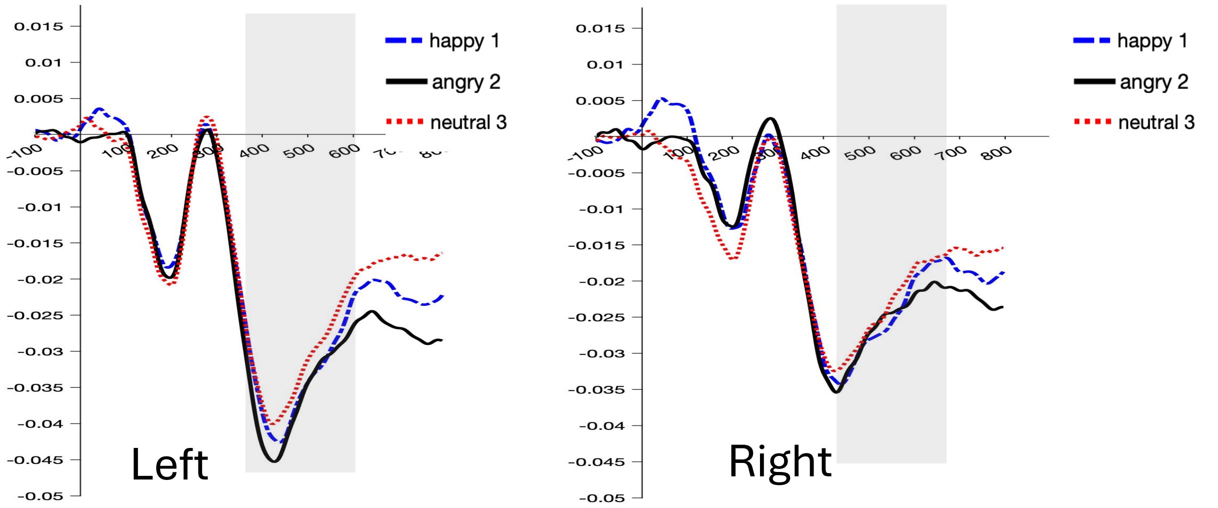

S4D.

Lower Risk (29 to 36 weeks)

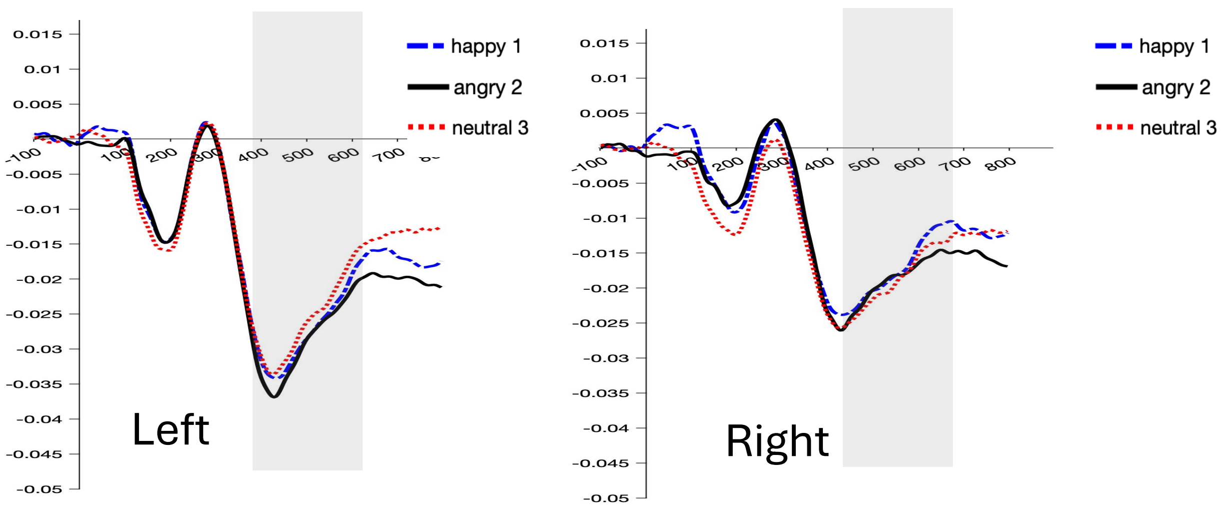

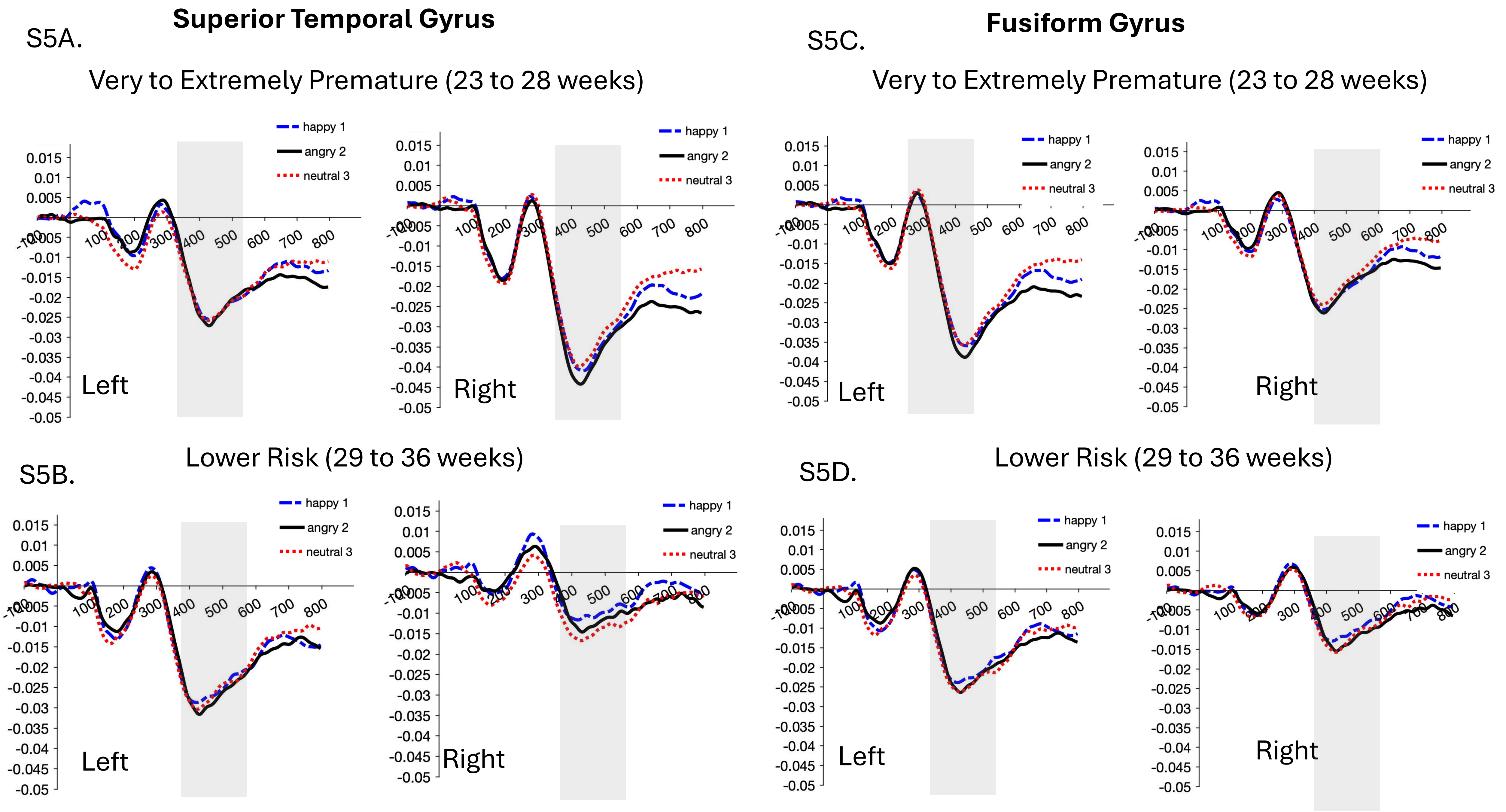

Superior Temporal Gyrus
Fusiform Gyrus
S5A.
Very to Extremely Premature (23 to 28 weeks)
S5B.
Lower Risk (29 to 36 weeks)
S5C.
Very to Extremely Premature (23 to 28 weeks)
S5D.
Lower Risk (29 to 36 weeks)
happy 1
angry 2
neutral 3
Left
Right

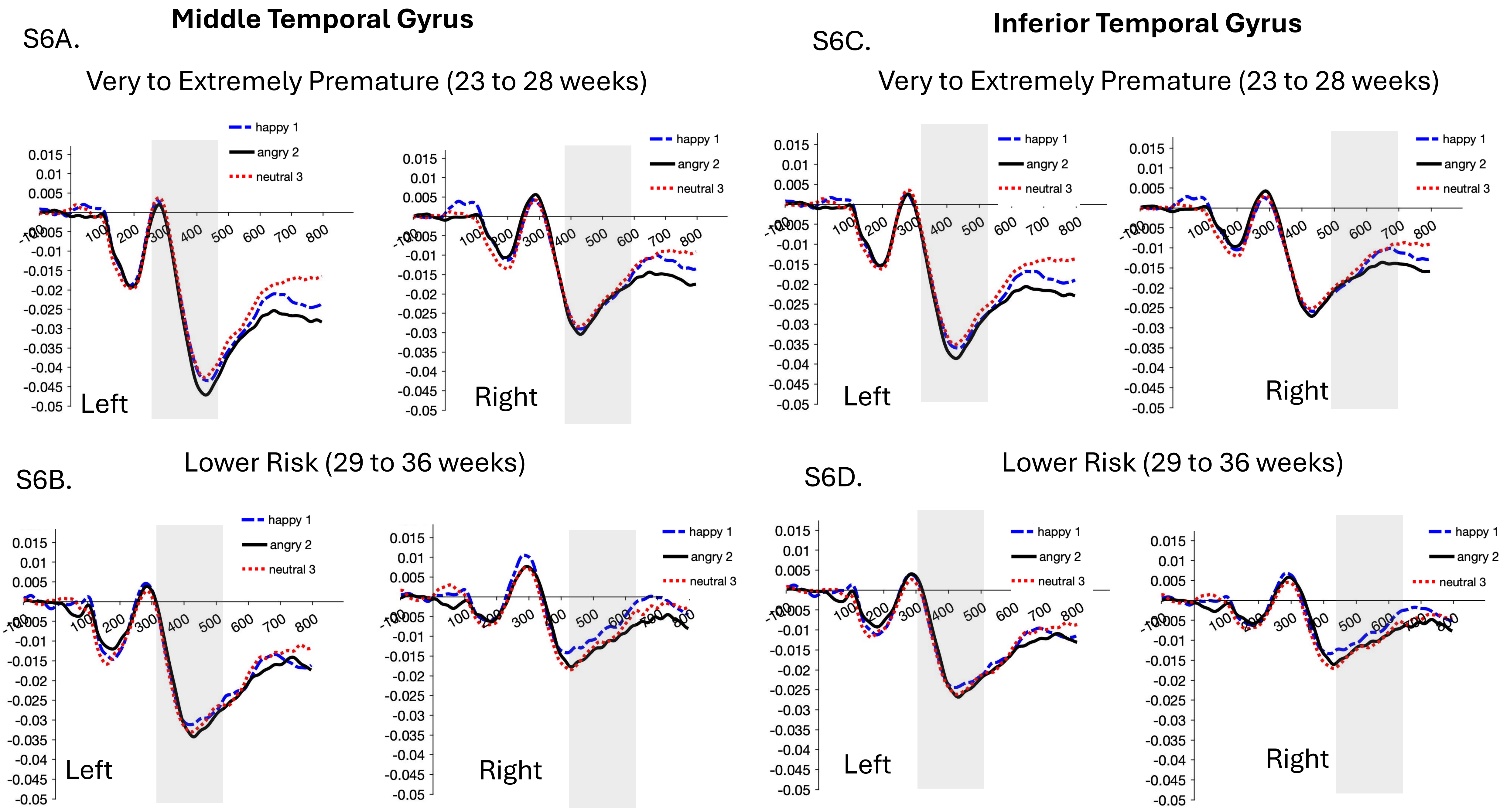
S6A.
Middle Temporal Gyrus
Very to Extremely Premature (23 to 28 weeks)
happy 1
angry 2
neutral 3
Left
Right
S6B.
Lower Risk (29 to 36 weeks)
happy 1
angry 2
neutral 3
Left
Right
S6C.
Inferior Temporal Gyrus
Very to Extremely Premature (23 to 28 weeks)
happy 1
angry 2
neutral 3
Left
Right
S6D.
Lower Risk (29 to 36 weeks)
happy 1
angry 2
neutral 3
Left
Right

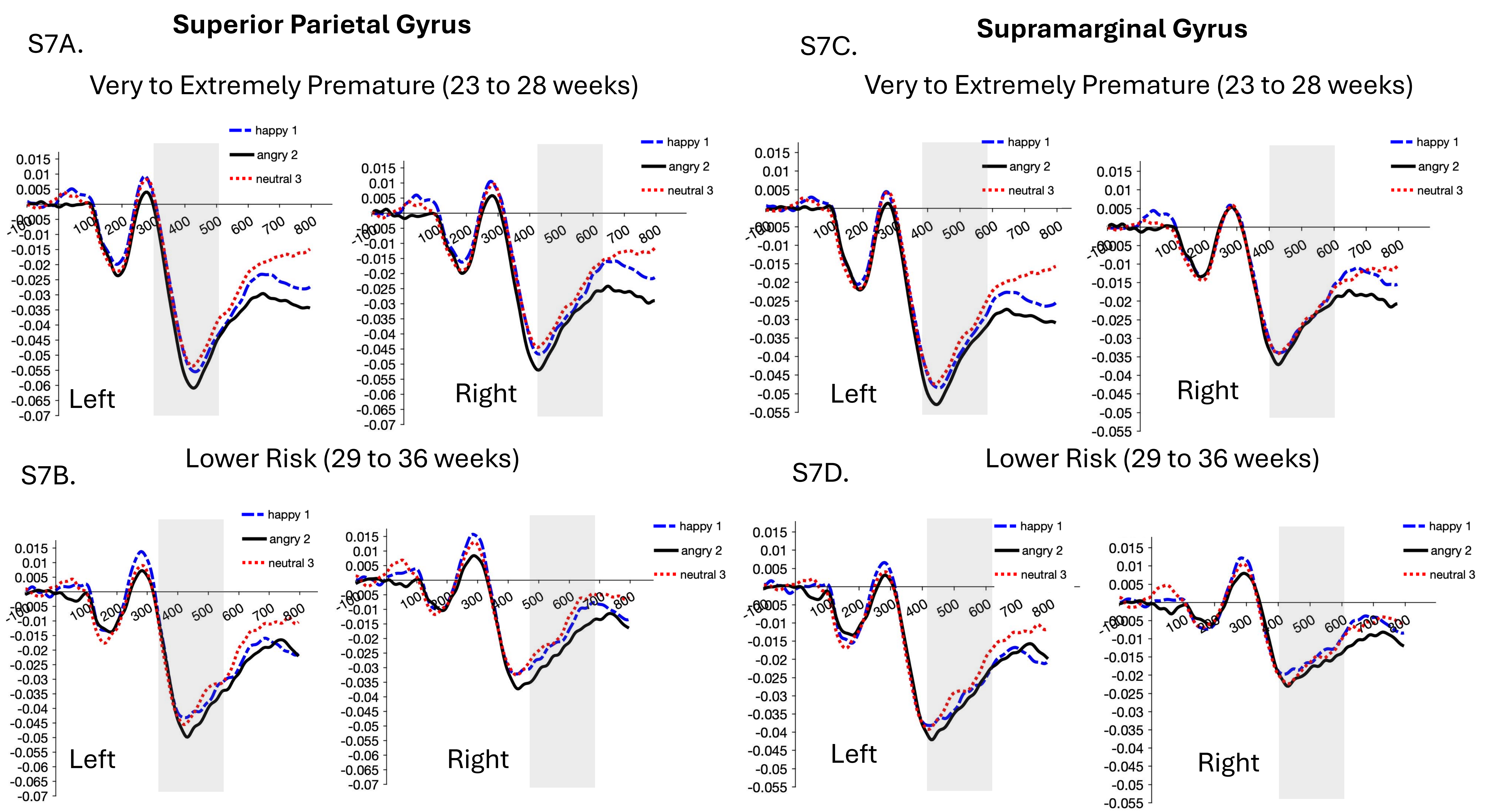
Superior Parietal Gyrus
S7A.
Very to Extremely Premature (23 to 28 weeks)
happy 1
angry 2
neutral 3
Left
Right
S7B.
Lower Risk (29 to 36 weeks)
Left
Right
Supramarginal Gyrus
S7C.
Very to Extremely Premature (23 to 28 weeks)
Left
Right
S7D.
Lower Risk (29 to 36 weeks)
Left
Right

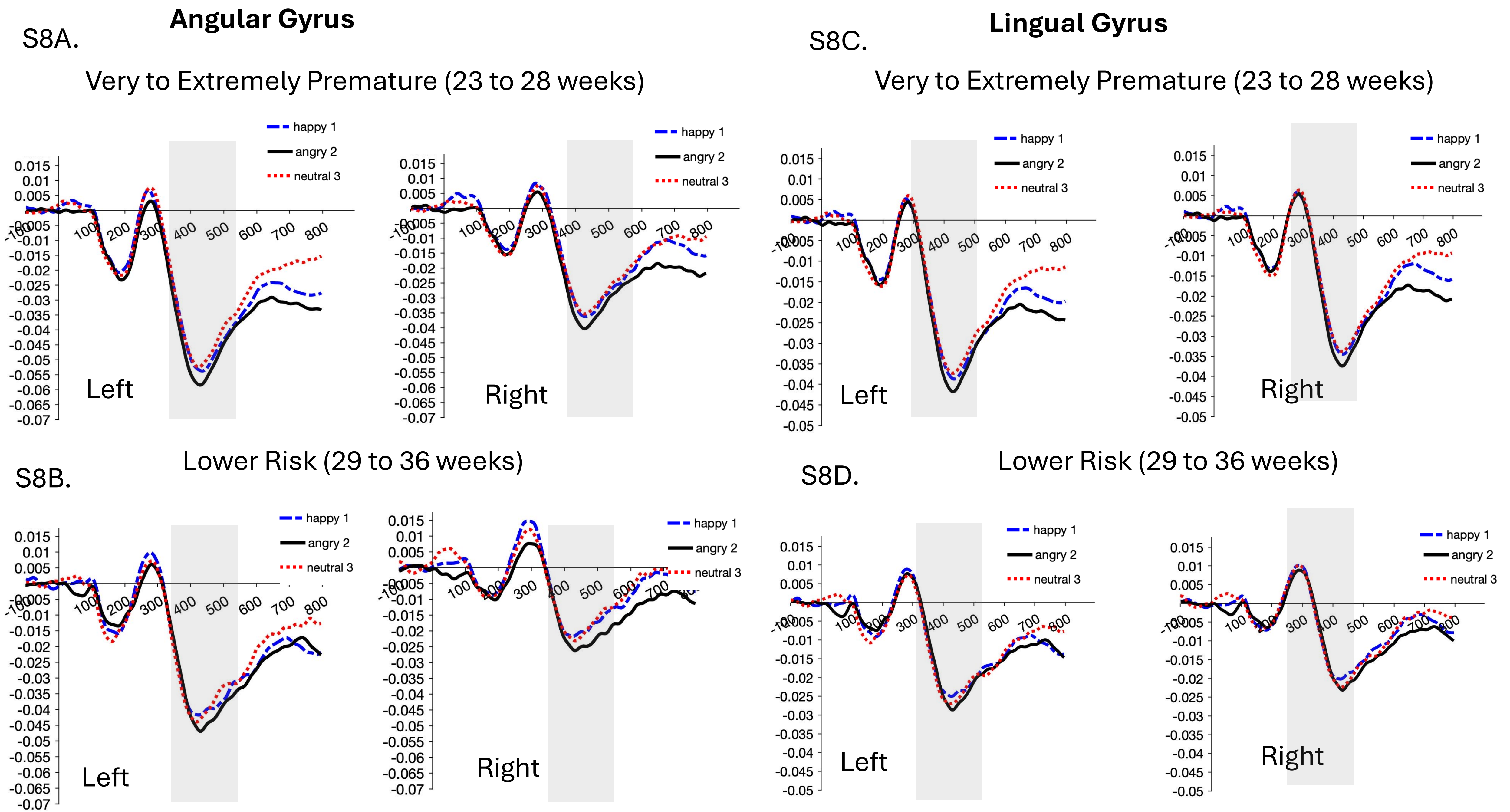
Angular Gyrus
S8A.
Very to Extremely Premature (23 to 28 weeks)
happy 1
angry 2
neutral 3
Left
Right
S8B.
Lower Risk (29 to 36 weeks)
happy 1
angry 2
neutral 3
Left
Right
Lingual Gyrus
S8C.
Very to Extremely Premature (23 to 28 weeks)
happy 1
angry 2
neutral 3
Left
Right
S8D.
Lower Risk (29 to 36 weeks)
happy 1
angry 2
neutral 3
Left
Right

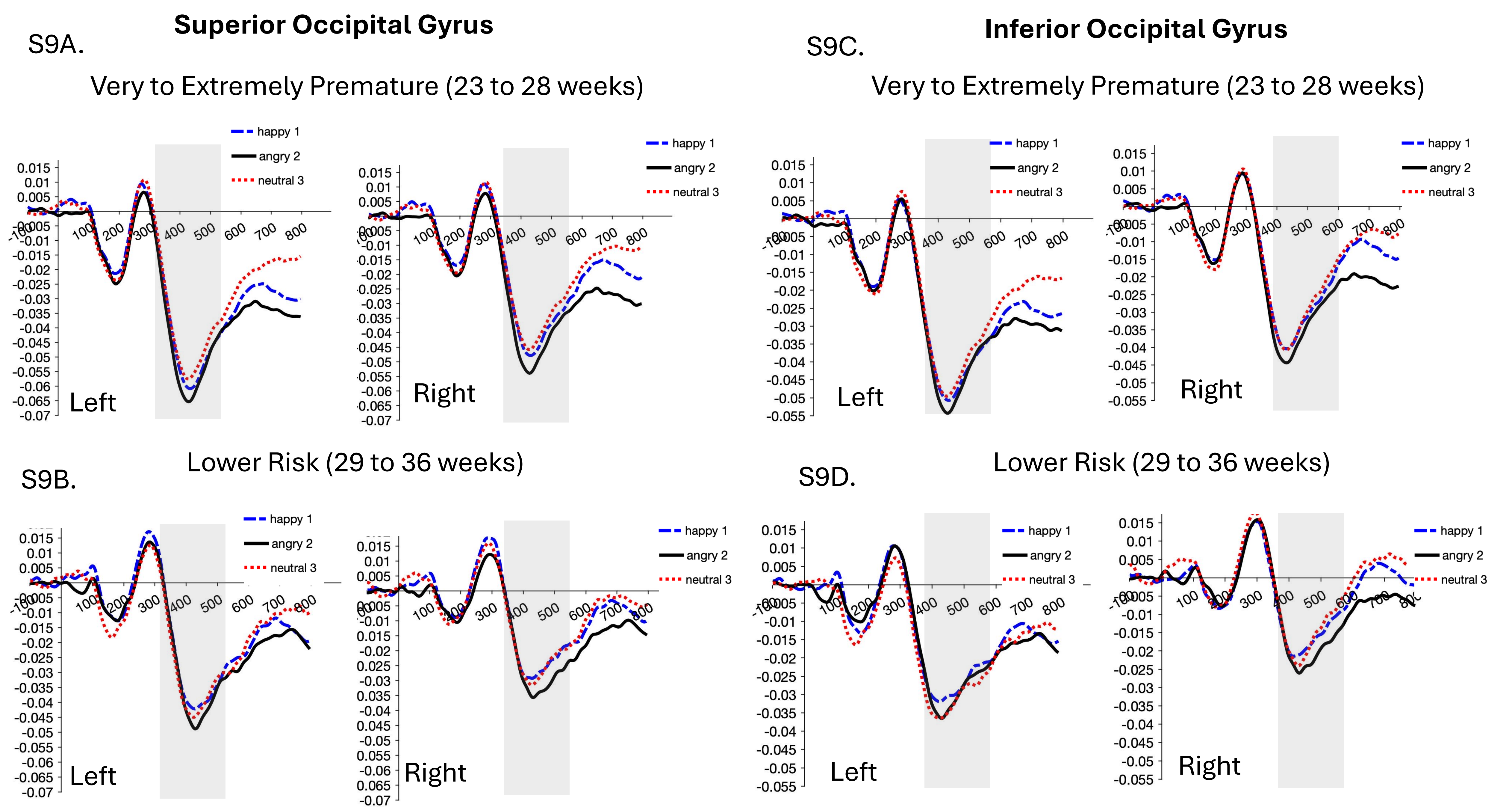
Superior Occipital Gyrus
S9A.
Very to Extremely Premature (23 to 28 weeks)
happy 1
angry 2
neutral 3
Left
Right
S9B.
Lower Risk (29 to 36 weeks)
Left
Right
Inferior Occipital Gyrus
S9C.
Very to Extremely Premature (23 to 28 weeks)
Left
Right
S9D.
Lower Risk (29 to 36 weeks)
Left
Right
